# A New Leptoceratopsid (Ornithischia, Ceratopsia) with a Unique Ischium from the Upper Cretaceous of Shandong Province, China

**DOI:** 10.1371/journal.pone.0144148

**Published:** 2015-12-23

**Authors:** Yiming He, Peter J. Makovicky, Kebai Wang, Shuqing Chen, Corwin Sullivan, Fenglu Han, Xing Xu

**Affiliations:** 1 Key Laboratory of Vertebrate Evolution and Human Origins, Institute of Vertebrate Palaeontology and Paleoanthropology, Chinese Academy of Sciences, Beijing, China; 2 Department of Geology, The Field Museum, Chicago, United States of America; 3 Zhucheng Dinosaur Museum, Zhucheng, Shandong, China; 4 School of Earth Sciences, China University of Geosciences, Wuhan, China; 5 University of Chinese Academy of Sciences, Beijing, China; Raymond M. Alf Museum of Paleontology, UNITED STATES

## Abstract

The partial skeleton of a leptoceratopsid dinosaur, *Ischioceratops zhuchengensis* gen. et sp. nov., was excavated from the bone-beds of the Upper Cretaceous Wangshi Group of Zhucheng, Shandong Province, China. This fossil represents the second leptoceratopsid dinosaur specimen recovered from the Kugou locality, a highly productive site in Zhucheng. The ischium of the new taxon is morphologically unique among known Dinosauria, flaring gradually to form an obturator process in its middle portion and resembling the shaft of a recurve bow. An elliptical fenestra perforates the obturator process, and the distal end of the shaft forms an axehead-shaped expansion. The discovery of *Ischioceratops* increases the known taxonomic diversity and morphological disparity of the Leptoceratopsidae.

## Introduction

The leptoceratopsids are a group of small, quadrupedal horned dinosaurs that have so far been found exclusively in the Upper Cretaceous (upper Santonian—upper Maastrichtian) of Asia and western North America [1]. With a typical body length of about two meters, they are much smaller than the contemporary ceratopsids [2]. The leptoceratopsids are characterized by robust jaws equipped with highly specialized large teeth and, unlike ceratopsids, lack horns and have extremely short frills [2, 3, 4]. Nevertheless, leptoceratopsids share some of the advanced features seen in ceratopsids and are closely related to the latter group.

Leptoceratopsidae was originally named by Nopcsa in 1923 [5] as a subfamily, with *Leptoceratops gracilis* as the type species. In 2001, Makovicky redefined Leptoceratopsidae as a stem-based taxon consisting of all species closer to *Leptoceratops gracilis* than to *Triceratops horridus* [6]. Leptoceratopsids were once known only from the Upper Cretaceous of North America [4,7], but three taxa have been described from the Upper Cretaceous of Asia: *Asiaceratops salsopaludalis* [8] from Uzbekistan, *Udanoceratops tschizhovi* [9] from Udan-Sayr, Mongolia, and *Zhuchengceratops inexpectus* [10] from the Kugou locality, Zhucheng, China [11]. Leptoceratopsids are a relatively basal clade within Neoceratopsia [3,6], whose success as a parallel radiation to Ceratopsidae has been demonstrated by several important discoveries over the past few decades, including that of *Prenoceratops pieganensis* [12], and *Cerasinops hodgskissi* [13].

Here we report a new leptoceratopsid dinosaur that was also excavated from the bonebeds of the Upper Cretaceous Wangshi Group of Zhucheng [14]. The new specimen, like *Zhuchengceratops*, comes from the Kugou locality. This locality, together with Longgujian (just 600 m north of Kugou) and Zangjiazhuang (5 km away from Kugou), has yielded numerous hadrosaurid bones [10]. The Zangjiazhuang locality has also produced several tyrannosaurid elements [15] and some material atrributable to *Sinoceratops zhuchengensis*, the only undisputed ceratopsid from outside of North America [16]. Though lacking cranial elements, the newly collected specimen possesses some morphological features that identify it as a non-ceratopsid neoceratopsian. In particular, the morphology of the ischium is unique among known Dinosauria. Discovery of this new taxon increases the taxonomic diversity and morphological disparity of the Leptoceratopsidae and has significant implications for interpretations of neoceratopsian biogeography.

## Materials and Methods

### Material

**Fig 1:**
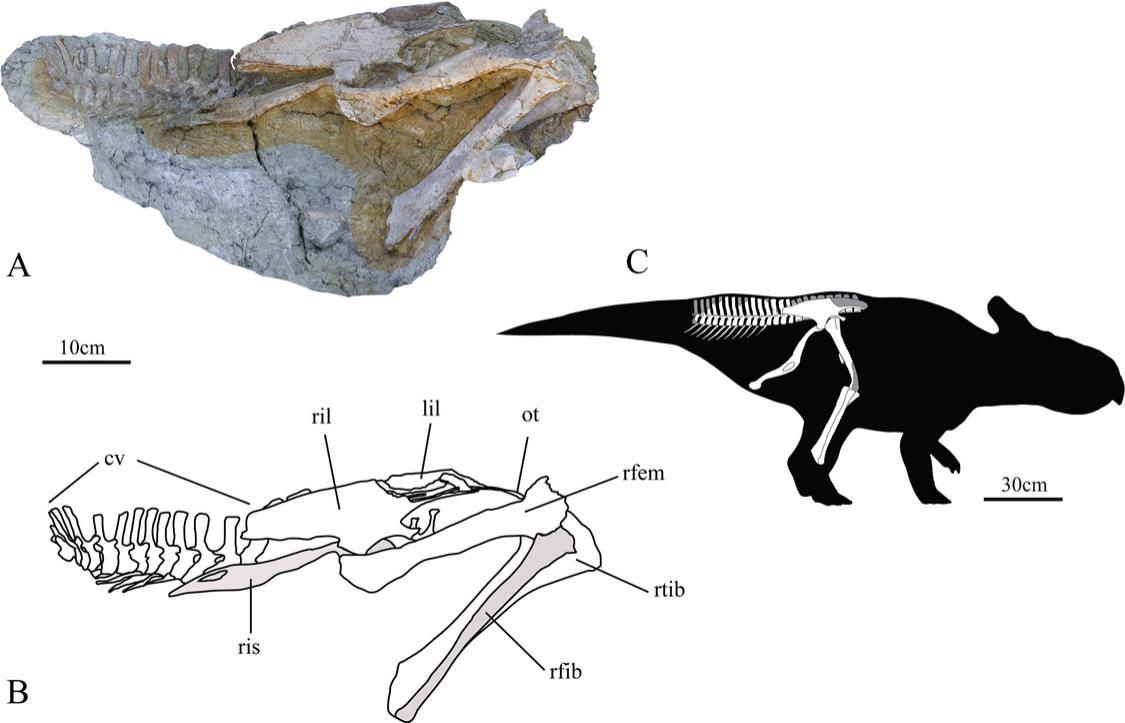
Holotype of *Ischioceratops zhuchengensis* (ZCDM V0016) in right lateral view. Photograph (A), drawing (B) and reconstruction of holotype individual (C). Abbreviations: cv, caudal vertebrae; lil, left ilium; ot, ossified tendons; rfem, right femur; rfib, right fibula; ril, right ilium; ris, right ischium; rtib, right tibia.

**Fig 2:**
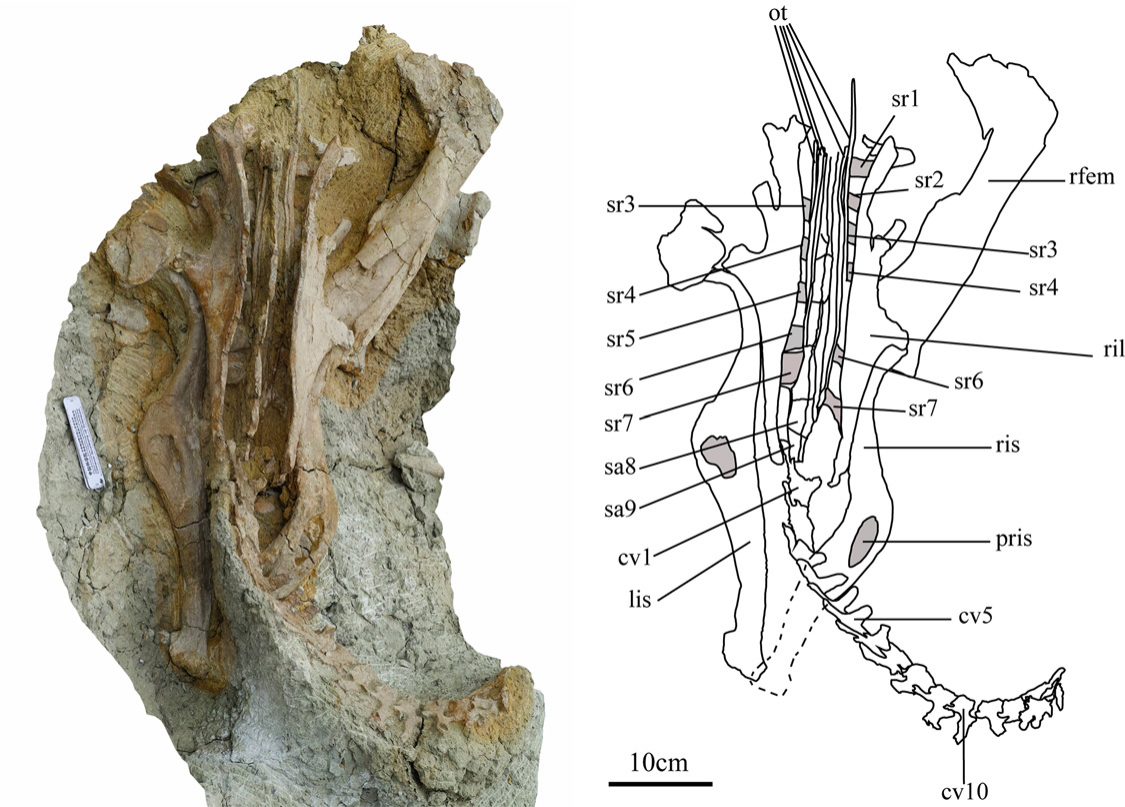
Holotype of *Ischioceratops zhuchengensis* (ZCDM V0016) in dorsal view. Photograph (left, scale bar equals 8 cm) and drawing (right). Abbreviations: cv, caudal vertebrae; lis, left ischium; ot, ossified tendons; pris, pit on right ischium; rfem, right femur; ril, right ilium; ris, right ischium; sa, sacral; sr, sacral rib.

**Fig 3:**
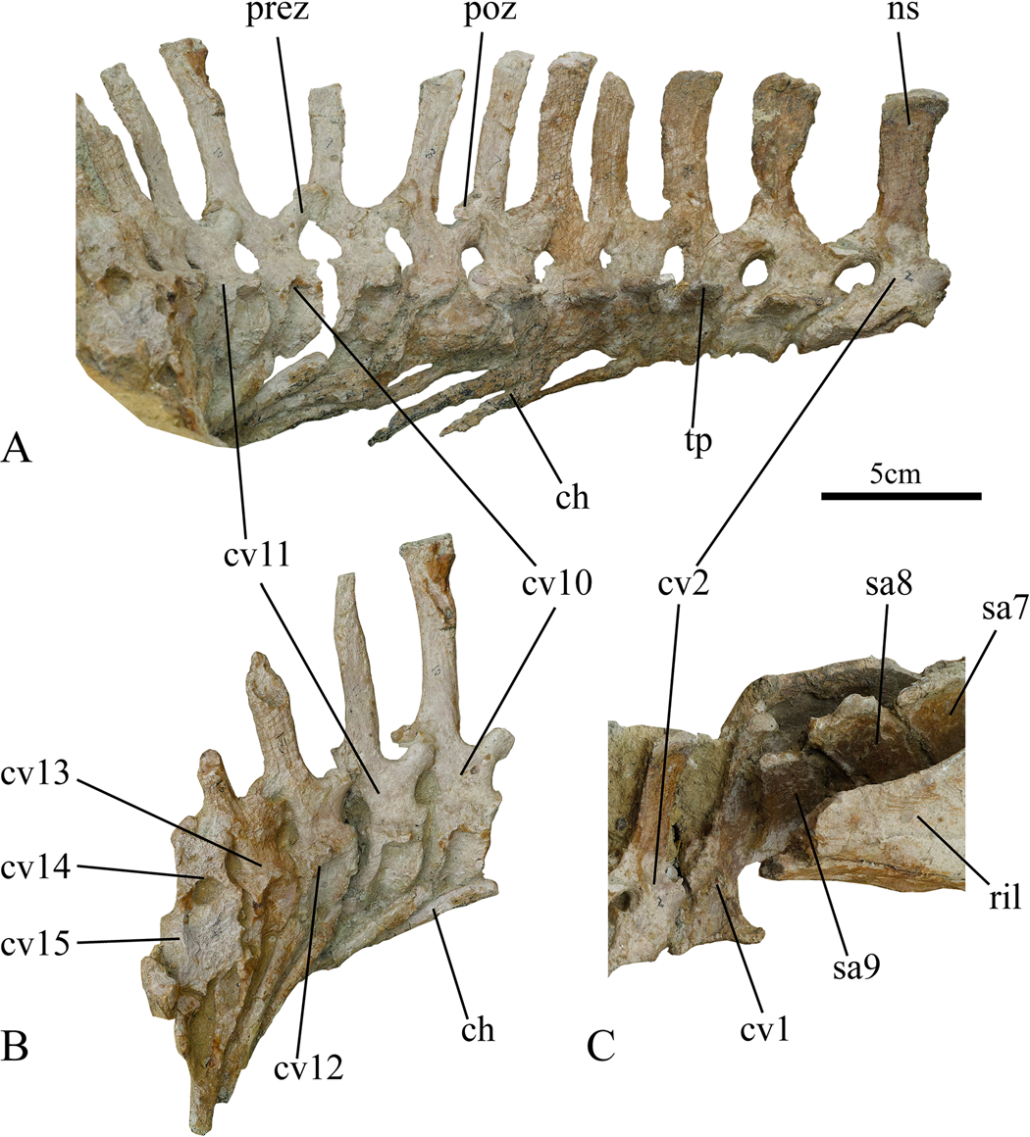
Caudal vertebrae of holotype of *Ischioceratops zhuchengensis* (ZCDM V0016) in right lateral view. Caudals 2–11 (A), caudals 10–15 (B), caudals 1–2 (C). Abbreviations: ch, chevron; cv, caudal vertebrae; ns, neural spine; poz, postzygapophysis; prez, prezygapophysis; ril, right ilium; sa, sacrals; tp, transverse process.

**Fig 4:**
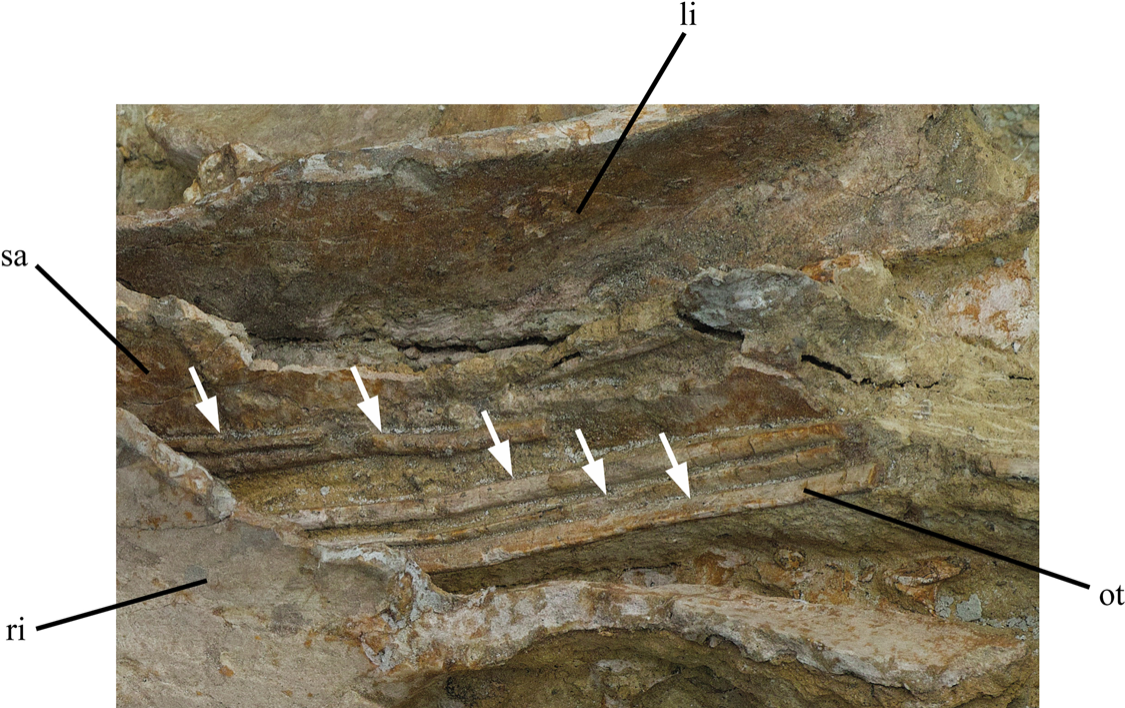
Ossified tendons of holotype of *Ischioceratops zhuchengensis* (ZCDM V0016), indicated by white arrows. Abbreviations: li, left ilium; ot, ossified tendons; ri, right ilium; sa, sacral vertebrae.

**Fig 5:**
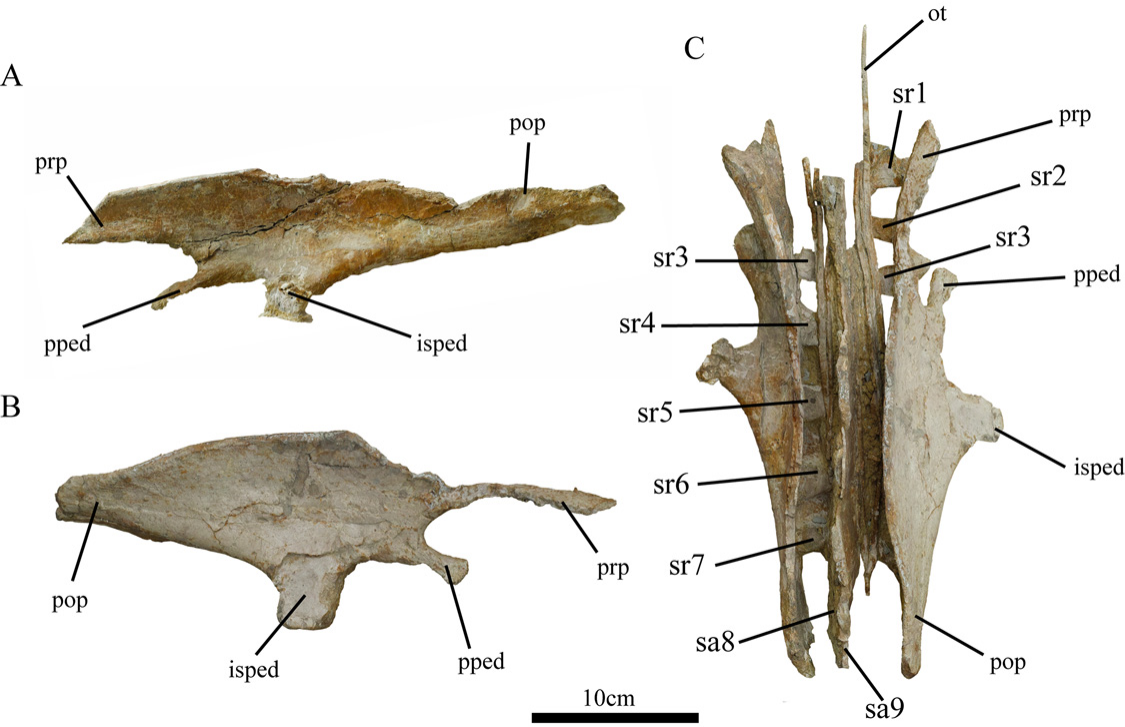
Pelvic girdle of holotype of *Ischioceratops zhuchengensis* (ZCDM V0016). Left ilium in lateral view (A), right ilium in lateral view (B), and both ilia in dorsal view (C). Abbreviations: isped, ischiadic peduncle; ot, ossified tendons; pop, postacetabular process; pped, pubic peduncle; prp, preacetabular process; sa, sacral vertebra; sr, sacral rib.

**Fig 6:**
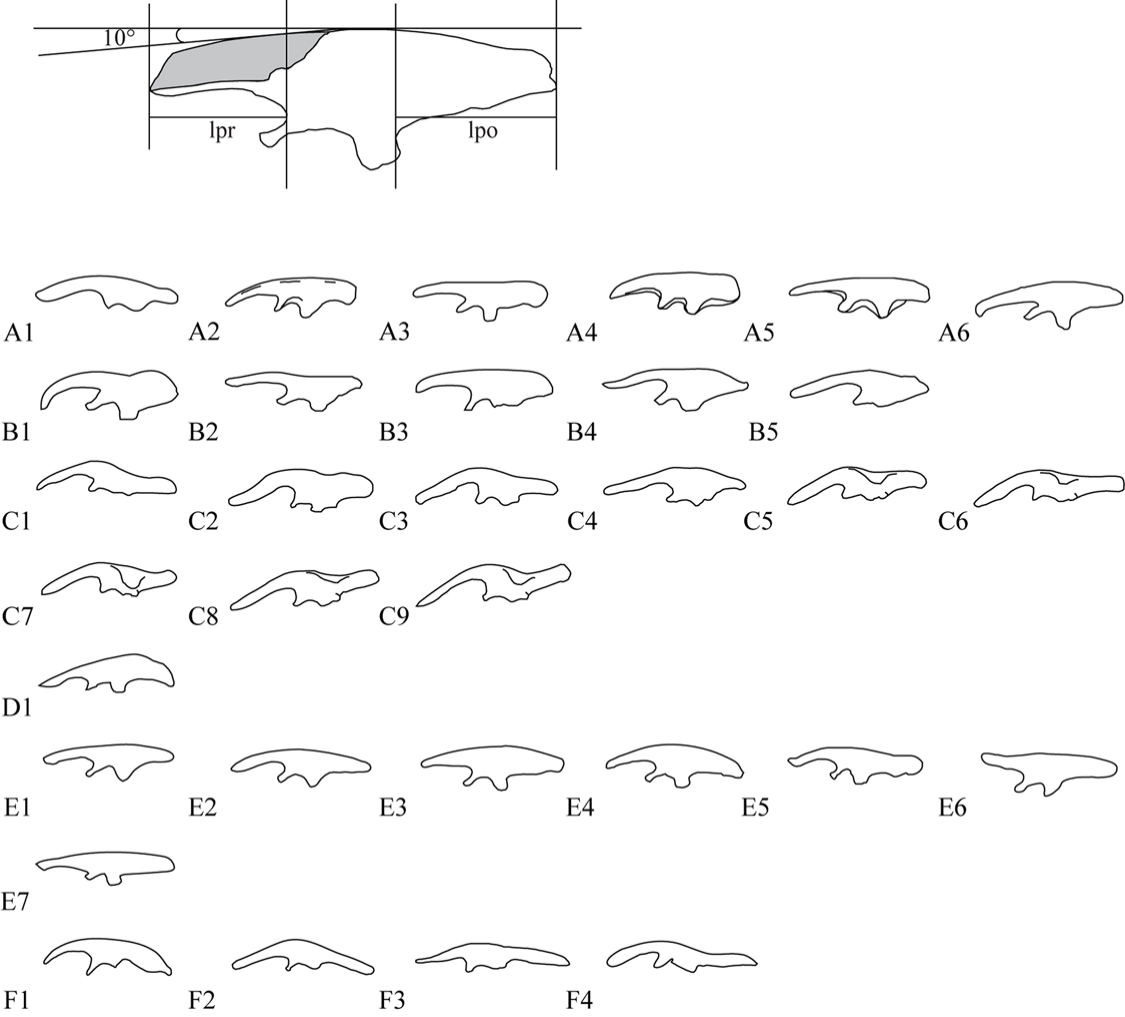
Ornithischian left ilia in lateral view. All figures are just outlines, similar but not identical to the original image. All figures are for illustrative purposes only. Outlines are not to scale. A1, *Heterodontosaurus tucki*, outlined from [45]; A2, *Othnielia rex*, from [46]; A3, *Hypsilophidon foxii*, outlined from [34]; A4, *Hexinlusaurus multidens*, based on ZDMT 6001, outlined from [47]; A5 *Agilisaurus louderbacki*, based on ZDMT 6011, outlined from [47]; A6, *Jeholosaurus shangyuanensis*, based on IVPP V15939, outlined from [20]; B1, *Tenontosaurus tilleti* outlined from [48]; B2, *Dryosaurus altus*, based on HNM dy II, outlined from [49]; B3, *Camptosaurus dispar*, outlined from [50]; B4, *Iguanodon atherfieldensis*, outlined from [50], 1990; B5, *Ouranosaurus nigeriensis*, outlined from [50], 1990; C1, *Gryposaurus incurvimanus*, outlined from [51]; C2, *Parasaurolophus cyrtocristatus*, based on FMNH P27393, outlined from [52]; C3, *Corythosaurus casuarius*, outlined from [53]; C4, *Gilmoreosaurus mongoliensis*, based on AMNH 6551, outlined from [52]; C5, *Brachylophosaurus canadensis*, based on MOR794, outlined from [54]; C6, *Edmontosaurus regalis*, based on ROM 5167, outlined from [51]; C7, *Saurolophus osborni*, based on AMNH 5220, outlined from [51]; C8, *Maiasaura peeblesorum*, based on MOR unnumbered, outlined from [54]; C9, *Kritosaurus navajovius*, based on TMM 42309–2, outlined from [54]; D1, *Homocephale calathocercos*, outlined from [21]; E1, *Yinlong downsi*, based on IVPP V18637; E2, *Psittacosaurus neimongoliensis*, based on IVPP 12-0888-2, outlined from [55]; E3, *Archaeoceratops oshimai*, based on IVPP V11114, outlined from [24]; E4, *Auroraceratops rugosus*, outlined from [29]; E5, *Protoceratops andrewsi*, outlined from [6]; E6, *Leptoceratops gracillis*, based on NMC 8889, outlined from [56]; E7, *Montanoceratops cerorhynchus*, based on AMNH 6466, outlined from [28]; F1, *Centrosaurus*, outlined from [57]; F2, *Chasmosaurus belli*, outlined from [58]; F3, *Triceratops horridus*, based on YPM 1821, outlined from [59]; F4, *Styracosaurus albertensis*, based on AMNH 5372, outlined from [60]; upper: *Ischioceratops zhuchengensis*, based on ZCDM V0016.

**Fig 7:**
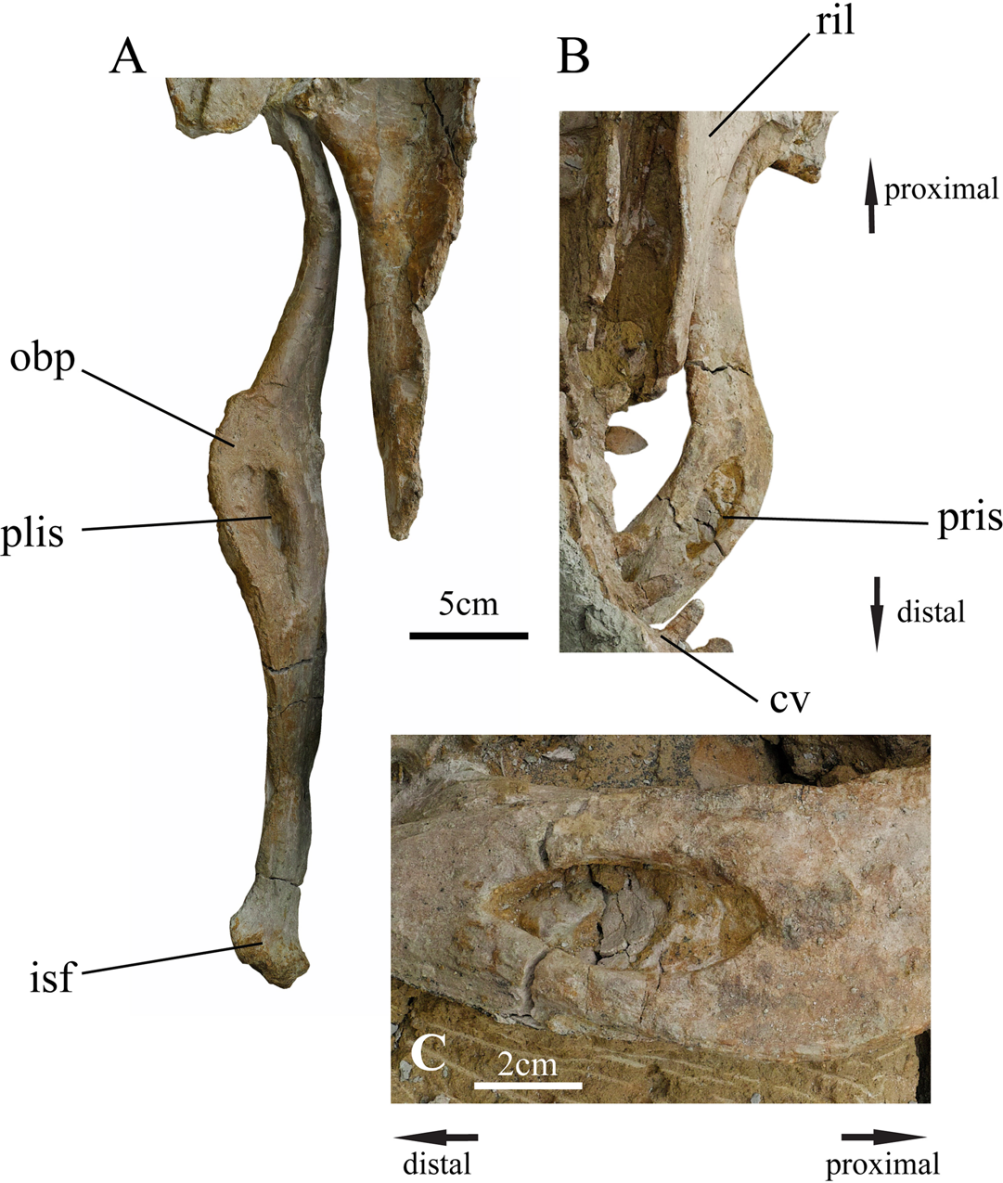
Ischia of holotype of *Ischioceratops zhuchengensis* (ZCDM V0016). Left ischium in lateral view (A); right ischium in lateral view (B); close-up of pit on the right ischium (C). Abbreviations: cv, caudal vertebrae; isf, ischiadic foot; obp, obturator process; plis, pit on left ischium; pris, pit on right ischium; ril, right ilium.

**Fig 8:**
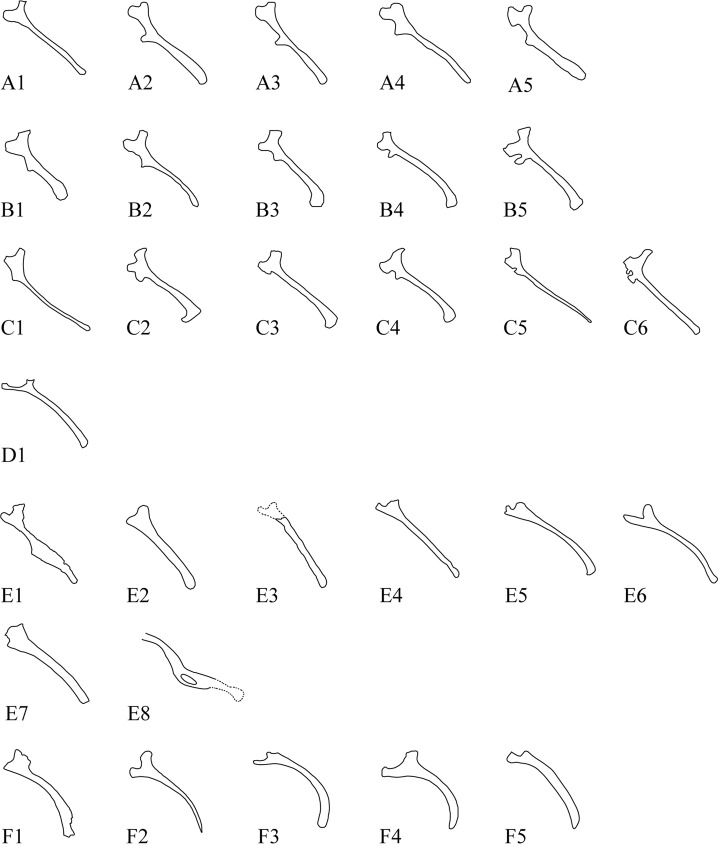
Ornithischian left ischia in lateral view. All figures are just outlines, similar but not identical to the original image. All figures are for illustrative purposes only. Outlines are not to scale. A1, *Heterodontosaurus tucki*, outlined from [45]; A2, *Othnielia rex*, outlined from [46]; A3, *Hypsilophodon foxii*, outlined from [34]; A4, *Hexinlusaurus multidens*, based on ZDM 6001, outlined from [47]; A5 *Agilisaurus louderbacki*, based on ZDMT 6011, outlined from [47]; B1, *Tenontosaurus tilleti*, outlined from [48]; B2, *Dryosaurus altus*, outlined from [49]; B3, *Camptosaurus dispar*, outlined from [50]; B4, *Iguanodon atherfieldensis*, outlined from [50]; B5, *Ouranosaurus nigeriensis*, outlined from [50]; C1, *Gryposaurus incurvimanus*, outlined from [51]; C2, *Parasaurolophus walkeri*, based on FMNH P27393, outlined from [52]; C3, *Corythosaurus casuarius*, outlined from [53]; C4, *Gilmoreosaurus mongoliensis* based on AMNH 6551, outlined from [52]; C5, *Edmontosaurus regalis* ROM 5167, outlined from [54]; C6, *Shantungosaurus giganteus*, outlined from [62]; D1, *Homocephale calathocercos*, outlined from [21]; E1, *Yinlong downsi*, based on IVPP V18679; E2, *Psittacosaurus neimongoliensis*, based on IVPP 12-0888-2, outlined from [55]; E3, *Archaeoceratops oshimai*, based on IVPP V11114, outlined from [24]; E4, *Auroraceratops rugosus*, outlined from [29]; E5, *Protoceratops andrewsi*, outlined from [4]; E6, *Leptoceratops gracillis*, based on NMC 8889, outlined from [56]; E7, *Montanoceratops cerorhynchus*, based on AMNH 6466, outlined from [42]; E8, *Ischioceratops zhuchengensis*, right and left ilium and ischium, based on ZCDM V0016; F1, *Centrosaurus apertus*, outlined from [57]; F2, *Chasmosaurus belli*, outlined from, [58]; F3, *Triceratops horridus*, based on YPM 1821, outlined from [59]; F4, *Styracosaurus albertensis*, based on AMNH 5372, outlined from [60].

The holotype of *Ischioceratops zhuchengensis* (ZCDM V00016 [Zhucheng Dinosaur Museum, Zhucheng, Shandong, China]) was excavated from Kugou, Zhucheng, Shandong Province, China; Upper Cretaceous Wangshi Group. The specimen was examined, measured, photographed at the Institute of Vertebrate Palaeontology and Paleoanthropology, Chinese Academy of Sciences, Beijing, China. *Ischioceratops zhuchengensis* is known exclusively from the holotype materials comprising an incomplete, partially articulated specimen (Figs 1–8) including the entire sacrum, a few ossified tendons, both halves of the pelvis, the anterior-most 15 caudal vertebrae in an articulated series, and the right femur, tibia and fibula.

### Nomenclatural acts

The electronic edition of this article conforms to the requirements of the amended International Code of Zoological Nomenclature, and hence the new names contained herein are available under that Code from the electronic edition of this article. This published work and the nomenclatural acts it contains have been registered in ZooBank, the online registration system for the ICZN. The ZooBank LSIDs (Life Science Identifiers) can be resolved and the associated information viewed through any standard web browser by appending the LSID to the prefix "http://zoobank.org/". The LSID for this publication is: urn:lsid:zoobank.org:pub: 19A423ED-8EAA -4842-9ECF-695876EC5EC0 and the LSID for the species is: urn:lsid:zoobank.org:pub:71CD0FAE-070C-4CC4-96CC-B37D5B1071CE. The electronic edition of this work was published in a journal with an ISSN, and has been archived and is available from the following digital repositories: PubMed Central, LOCKSS.

### Ethic statements

According to the legislation of the People’s Republic of China, all necessary permits were obtained for the described field studies from the Shandong Provincial Department of Land and Resources and from the Zhucheng Dinosaur Museum.

## Results

### Systematic palaeontology

Ornithischia Seeley, 1888 [17]

Ceratopsia Marsh, 1890 [18]

Leptoceratopsidae Nopcsa, 1923 [5]

*Ischioceratops zhuchengensis* gen. et sp. nov.

urn:lsid:zoobank.org:pub:71CD0FAE-070C-4CC4-96CC-B37D5B1071CE

#### Etymology

Genus name from ischium and ceratops (horn-face, Latinized Greek), in reference to the unique morphology of the ischium. The species name is in honor of Zhucheng, where the holotype specimen was discovered.

#### Holotype

Zhucheng Dinosaur Museum (ZCDM) V0016, an incomplete, partially articulated specimen (Figs 1–8) comprising the entire sacrum, a few ossified tendons, both halves of the pelvis, the anteriormost 15 caudal vertebrae in an articulated series, and the right femur, tibia and fibula.

#### Type locality and horizon

Kugou, Zhucheng, Shandong Province, China; Upper Cretaceous Wangshi Group [11].

#### Diagnosis

The specimen can be referred as a basal ceratopsian and distinguished from other known Dinosauria based on the following combination of characters: ossified tendons confirme that the specimen belongs to an ornithischian dinosaur, nine sacral vertebare exclude it from basal Ornithopoda or Anklysauridae, the lateral outline of ilium without lateral everted shelf on the dorsal edge exclude it from Iguanodontidae, Hadrosauridae, and Ceratopsidae. The neural spines of proximal caudals increase in length towards middle part of tail as in several basal ceratopsian dinosaurs such as *Koreaceratops*, *Protoceratops*, *Cerasinops* and *Montanoceratops*.

The specimen can be referred to Leptoceratopsidae and distinguished from other known leptoceratopsids based on the following combination of characters: nine sacral vertebrae, more than in any other known basal (non-ceratopsid) ceratopsian but fewer than in ceratopsids; The ischium is unique and presumably autapomorphic, with a robust shaft that resembles that of a recurved bow and flares gradually to form a subrectangular-shaped obturator process in its middle portion. An elliptical fenestra perforates the obturator process.

### Description

#### Sacral vertebrae

The sacral vertebrae are visible almost exclusively in dorsal view, being still largely buried in matrix (Fig 2). The sacrum appears to be composed of nine sacral vertebrae in tight natural articulation. The presence of six sacrals is the primitive cerapodan condition, and is retained in taxa such as *Psittacosaurus* [19], *Jeholosaurus* [20] and Pachycephalosauria [21]. More than six sacral vertebrae are present in derived ornithopods [22] and ceratopsians [23]. In previously known basal neoceratopsians, the number of sacral vertebrae varies from six to eight. There are six sacrals in *Archaeoceratops oshimai* [24] and *Leptoceratops gracilis* [25], seven in *Graciliceratops mongoliensis* [26, 27], and eight in *Protoceratops andrewsi*, *Montanoceratops* [28] and *Auroraceratops rugousus* [29]. But it is also should be noted that the sacral fusions varies between individuals and ontogeny [28, 29]. In ceratopsids, the sacrum consists of ten variably ankylosed centra [23]. The presence of nine sacrals suggests that *Ischioceratops* has a higher number of sacrals than has ever been previously reported for a basal ceratopsian.

The sacral ribs are trapezoidal in dorsal view, dorsoventrally compressed, and contact the ilium. The left rib of Sa1 has been lost. In dorsal view, the rib of Sa1 is the longest in the mediolateral dimension, those of Sa6 and Sa7 are shorter, and the other sacral ribs have intermediate, subequal lengths.

All of the sacral neural spines are incompletely preserved, but they are broad, robust and inclined caudodorsally. Although the neural spines are tightly appressed to one another, they appear to be unfused because the lines of contact between adjacent spines are quite clear. The spines are also unfused with each other in other basal ceratopsians [4] such as *Leptoceratops gracilis* [28] and *Auroraceratops rugosus* [29].

#### Caudal vertebrae

The specimen preserves the cranial-most 15 caudal vertebrae in articulation, forming a continuous series with the sacrals. The tail is exposed in right lateral view, with the left lateral side still buried in matrix. Ca13-14 are severely eroded, with most of the neural spines obliterated in each case, and very little of Ca15 remains (Fig 3B). The length of each centrum is nearly twice its height, and the heights of the centra increase gradually in the distal direction (Table 1), indicating that a large portion of the tail is missing. Complete specimens of *Leptoceratops* preserve between 38 and 48 caudals [25], while *Koreaceratops* has more than 36 [30]. These values suggest that more than half of the tail is missing in *Ischioceratops*.

**Table 1:** Measurements of the caudal vertebrae of the holotype specimen of *Ischioceratops zhuchengensis* (ZCDM V0016).

	Neural Spine Height (cm)	Centrum Height (cm)	Chevron Height (cm)
Ca1	6.4	2.1	/
Ca2	6.4	2.1	/
Ca3	7.0	2.2	/
Ca4	6.9	2.0	/
Ca5	6.7	2.2	4.9
Ca6	6.6	2.2	6.2
Ca7	6.9	2.3	6.2
Ca8	7.1	2.3	6.1
Ca9	5.6	2.3	5.1
Ca10	7.6	2.2	6.0
Ca11	7.1	2.0	6.5
Ca12	5.9	1.9	5.5
Ca13	3.9	2.0	6.1
Ca14	/	/	5.6
Ca15	/	/	/

The neural spines of the preserved caudal vertebrae are well developed. They are subrectangular in lateral view, tall, and laterally compressed. The neural spines of Ca1-3 are much thicker transversely than those of the subsequent caudals. The cranialmost three neural spines are slightly inclined caudally, whereas the others are vertical. The spines of Ca10-11 are the tallest preserved in the specimen (Fig 3A, Table 1), and the following ones are distinctly shorter. The heights of the spines of Ca10-11 are more than 3.5 times the lengths of the corresponding centra (Table 1). Many derived ornithischian dinosaurs exhibit neural spines that are much taller than the centra are long, but the highest ratios are observed in leptoceratopsid taxa [27].

*Ischioceratops* clearly resembles other basal neoceratopsians in that neural spine height increases posteriorly in the proximal part of the tail [4]. *Auroraceratops*, *Koreaceratops*, *Montanoceratops* and *Protoceratops* each have a “leaf-shaped” tail [4] in lateral view, the neural spines initially increasing in height but subsequently decreasing toward the end of the tail [4]. In *Koreaceratops*, the caudal vertebrae neural spines increase continuously to caudal 22, and this vertebrate is approximately 5.6 times as high as the vertebral centrum [30]. In *Auroraceratops*, the neural spines increase in height, and the tallest preserved one is nearly three times as high as the corresponding centrum [29]. In *Montanoceratops*, the tallest neural spine (Ca15) occurs in the middle of the tail and is approximately four times as high as the corresponding centrum [30]. In *Protoceratops*, the neural spines increase in height until Ca14, about one-third of the distance from the base of the tail to the tip, and then decrease to the end of the tail [4].

The prezygapophyses are much longer and broader than the postzygapophyses, which are positioned below the caudal edges of the neural spines. The pre- and postzygapophyses extend a little beyond the anterior and posterior ends of the centra, respectively, and the postzygapophyses are positioned higher on the neural arches than the prezygapophyses. Most of the transverse processes are broken; only their bases remaining intact. They have elliptical cross-sections at the base.

Eleven chevrons are preserved in the specimen. The first chevron is situated between Ca4 and Ca5, articulating with the ventral surfaces of the centra of these vertebrae. In lateral view, the chevrons have a simple rod-like appearance and are inclined strongly posteriorly, making an angle of nearly 30° with the long axis of the tail. The proximal end of each centrum appears wedge-shaped in lateral view. The distal tips of the chevrons appear to be broken, so that their original lengths (Table 1) may have slightly exceeded their current, preserved lengths. As preserved, the chevrons are slightly shorter than the corresponding neural spines, a condition seen in intact caudals of some derived ornithopods such as *Ouranosaurus* and *Edmontosaurus* as well as some basal ceratopsians such as *Koreaceratops*, *Montanoceratops*, *Udanoceratops*, and *Protoceratops* [30]. In most ceratopsians, however, the chevrons are about as long as the corresponding neural spines [4,23].

#### Ossified tendons

The epaxial ossified tendons run longitudinally along the lateral surfaces of the neural spines of the sacral vertebrae and terminate abruptly at the boundary between the neural spines of the last sacral and the first caudal (Fig 2). They are long, slender, and round in cross section, and appear to be parallel with each other Fig 4). The distribution of this type of ossified tendon is similar to that in the basal ornithischians *Lesothosaurus diagnosticus* [31], *Agilisaurus louderbacki* [32], some ornithopods (e.g., *Jeholosaurus* [20]; *Haya griva* [33]), some basal ceratopsians and ceratopsids [4,23]. In other ornithopods such as *Hypsilophodon foxii* [34] and *Tenontosaurus tilleti* [35], as well as in some *Psittacosaurus* species (e.g., *P*. *xinjiangensis*, [19]) and other basal ceratopsians [4], ossified tendons extend along at least the proximal half of the caudal series. In iguanodontians, including hadrosaurids, the ossified tendons are organized into a rhomboidal lattice along the dorsal side of the vertebral column [36–40].

#### Ilium

Both ilia are present, but are imperfectly preserved. The ventral and medial surfaces of the ilia are buried in the matrix. The ilium consists of a main body, a robust preacetabular process, an elongate postacetabular process, a short and slim pubic peduncle and a quadrangular ischiadic peduncle. The central portion of the right preacetabular process (Fig 5B) and the upper portion of the left postacetabular process (Fig 5A) are broken.

The ilium is craniocaudally elongate, with a slightly concave lateral surface. The ilium resembles those of other basal neoceratopsians and those of basal ornithopods in being dorsoventrally tall and having a convex dorsal margin (Fig 6A1–6A6, 6D1 and 6E1–6E7). The anterior portions of both ilia curve laterally due to the deflection of the preacetabular processes, whereas their caudalmost portions are medially deflected (Fig 5C). Similar sigmoid curvature in dorsal aspect is observed in the ilia of *Protoceratops* [41]. The lack of strong eversion of the dorsal margin of the iliac blade distinguishes *Ischioceratops* from iguanodontians (Fig 6B1–6B5), *Pachycephalosaurs* (Fig 6D1) and ceratopsids (Fig 6F1–6F4). However, *Protoceratops* (Fig 6E6), *Montanoceratops* (Fig 6E7, [42]) and *Leptoceratops* are more similar to *Ischioceratops* in that the iliac blade is only slightly everted in these taxa.

The cranial tip of the preacetabular process is missing on both sides of the skeleton (Fig 5A and 5B). However, the preserved length of the preacetabular process accounts for approximately 35% of the total length of the ilium. The ventral border is thickened and strongly deflected medially, giving the process an L-shaped cross-section. The preacetabular process is inclined anteroventrally at about 10° to the horizontal (Fig 6, top).

The postacetabular process is well preserved in the right ilium (Fig 5B). The postacetabular process is slightly longer than the preacetabular process, accounting for nearly 39% of the length of the ilium, and is also considerably dorsoventrally deeper than the preacetabular process in lateral view. The approximate equality in length between the postacetabular and preacetabular processes (Fig 6, top) is shared by *Psittacosaurus* (Fig 6E2, [4, 43]) and other ceratopsians (Fig 6E1–6E7). The postacetabular process narrows gradually as it extends caudally, as in *Leptoceratops* (Fig 6E6, [26]). The caudal tips of both ilia are eroded. The lateral surface of the postacetabular process is generally smooth. The ventral border is deflected medially, like that of the preacetabular process, but only to a slight degree.

The anteroventral part of the ilium consists of two processes that arise from the central plate: a ventrally extending ischiadic peduncle and an anteriorly extending pubic peduncle. The pubic peduncle is exceptionally slender and strongly compressed dorsoventrally. It forms an angle of approximately 30° with the preacetabular process in lateral view. The pubic peduncle is slightly deflected laterally. The pubis is not visible, but may be buried in the matrix ventral to the peduncle. The large and robust ischiadic peduncle is vertically oriented, and appears rectangular in lateral view. The peduncle is similar in shape to the condition in *Auroraceratops* (Fig 6E4), *Protoceratops* (Fig 6E5) and *Montanoceratops* (Fig 6E7), but different from *Archaeoceratops* (Fig 6E3), *Yamaceratops* [44] and *Leptoceratops* (Fig 6E6) which had a tapered distal end.

#### Ischium

Both ischia are completely preserved but exposed only in lateral view (Fig 7). The shape of the ischium is the most significant feature of the present specimen, unique among known dinosaurs.

The left ischium measures approximately 36.2 cm in length. Although it has undergone taphonomic distortion, its curvature is less pronounced than that of the right ischium. The angle between the ischium and the ilium is uncertain, owing to preservational distortion. The shaft is long and mediolaterally compressed, with a subrectangular cross-section. On each side of the specimen, the proximal portion of the ischium remains in place beneath the ischiadic peduncle of the ilium, so the exact shapes of the proximal peduncles for articulation with the pubis and ilium are still unknown. In lateral view, the shaft resembles that of a recurved bow, in which the distal tips of the limbs curve away from the archer when the bow is unstrung (Fig 7A). The middle portion of the shaft flares gradually to form a subrectangular-like obturator process (Fig 7A and 7B). The obturator process differs in shape from the superficially similar structure that occurs in some basal ornithischian dinosaur and ornithopod dinosaurs (Fig 8A2–8C6), which projects abruptly from the ventral border of the shaft close to its proximal end. No obturator process is present on the ischium in other ceratopsians (Fig 8E1–8F4, [4, 23]), or in *Pachycephalosaurs* (Fig 8D1, [22]). (Though the ischium in *Yinlong* is quite different from the other ceratopsian ischia in lateral view, it is not the same as *Ischioceratops* or some hadrosaurids. The shaft of the right ischium of *Yinlong* (Fig 8E1, from IVPP V18679) is “bowie-knife” shaped in lateral view. The cross-section of the shaft is quite thin just like a blade. No “obturator-like” structure as in hadrosaurid or *Ischioceratops* is present in *Yinlong*.

The proximal portion of the shaft is mediolaterally compressed, and its lateral surface bears a shallow longitudinal groove. A large elliptical pit, which tapers toward both its cranial and caudal ends, occurs on the “obturator process”. In the left ischium the pit is strongly compressed, apparently due to taphonomic distortion, but in the right ischium the pit is better preserved. The length and height of the pit in the right ischium are 5.5 cm and 2.1 cm, respectively. The pit is deepest at its center, and becomes shallower toward both its proximal and distal ends. However, the proximal end of the pit slopes steeply, and the distal end more gently (Fig 7C). The pit probably represents a site of muscle attachment. In the basal ornithischian *Lesothosaurus*, Maidment and Barrett (2011) [61] reconstructed two slips of the femoral adductor musculature as originating on the ventral and dorsal edges of the ischial shaft, and placed the insertion of the puboischiofemoralis externus between them. These muscles may have been associated with the unusual expansion and pit on the ischial shaft of *Ischioceratops*

The caudal part of the shaft curves gently caudoventrally. The distal end of the shaft is dorsoventrally expanded to form a large semicircular ischial “foot”, with a rugose surface texture. The distal ends of the ischia contact one another below Ca4 and Ca5 (Fig 2).

#### Femur

**Fig 9:**
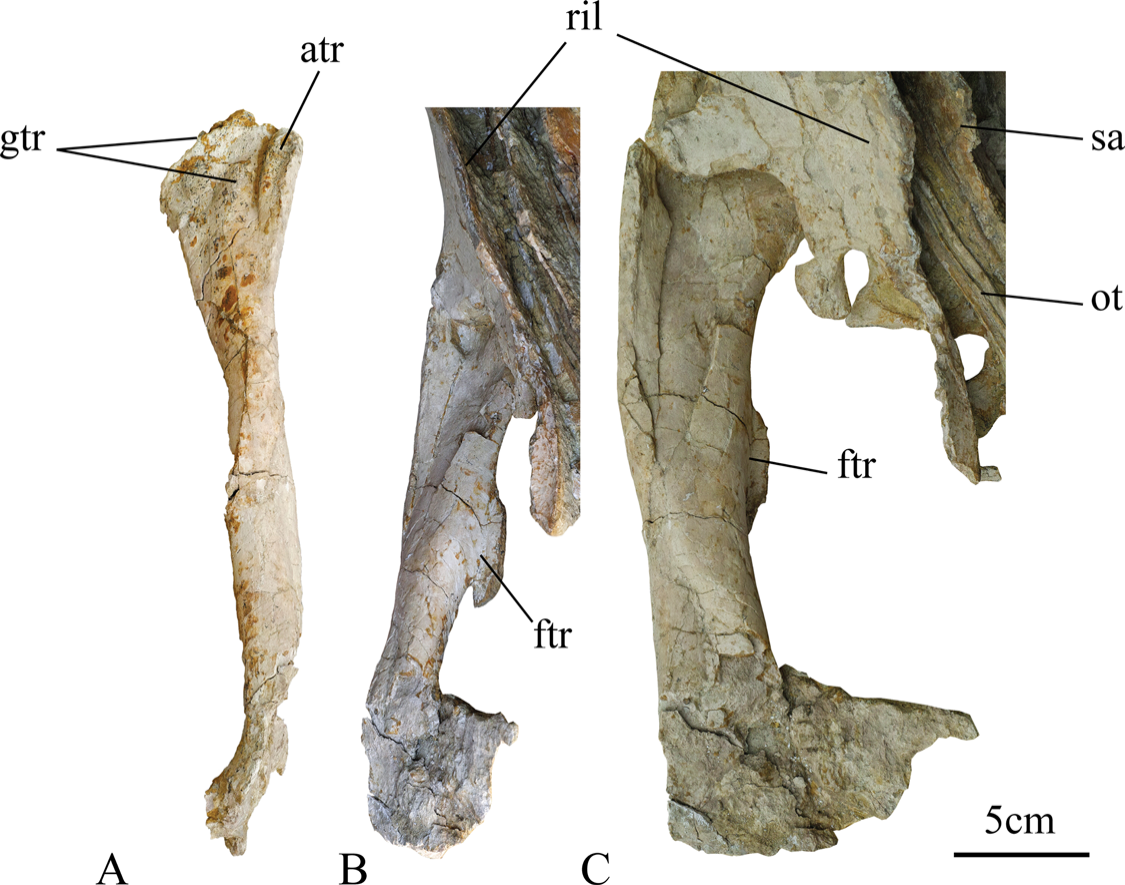
Right femur of holotype of *Ischioceratops zhuchengensis* (ZCDM V0016). Right femur in lateral (A), medial (B) and anterior views (C). Abbreviations: atr, lesser trochanter; ftr, fourth trochanter; gtr, greater trochanter; ot, ossified tendons; ril, right ilium; sa, sacrals.

The right femur is almost complete, although the distal end is eroded (Figs 1, 2 and 9). Only a part of the proximal end of the left femur is preserved (Fig 2).

The right femur is approximately 29.1 cm long, and has a transverse midshaft diameter of nearly 44.1 mm. The posterior side of the femur is still buried in the matrix (Fig 2). The anterior surface of the femoral shaft is crushed to form a concavity, and this surface has been damaged by erosion near the distal end of the femur.

The femoral shaft is straight in anterior view, and bowed slightly anteriorly in lateral view. The shaft is elliptical in cross-section. The proximal end is covered by the pelvic girdle, with which it remains in articulation.

The greater trochanter is expanded anteroposteriorly. The lesser trochanter is narrow and closely appressed to the greater trochanter, from which it is separated by a deep notch visible in lateral view. The notch may be a primitive character among ornithischian dinosaurs, because it occurs in several basal ornithischians and ornithopods including *Hexinlusaurus multidens* [63], *Abrictosaurus consors* [64], *Dryosaurus altus* [49], *Camptosaurus dispar* [65] and *Eocursor parvus* [66, 67]. In other ornithopods, such as *Jeholosaurus* [20], and in ceratopsians such as *Psittacosaurus* [19], there is only a faint crease on the surface where the two trochanters contact one another. The lesser trochanter does not extend quite as far proximally as the greater trochanter.

**Fig 10:**
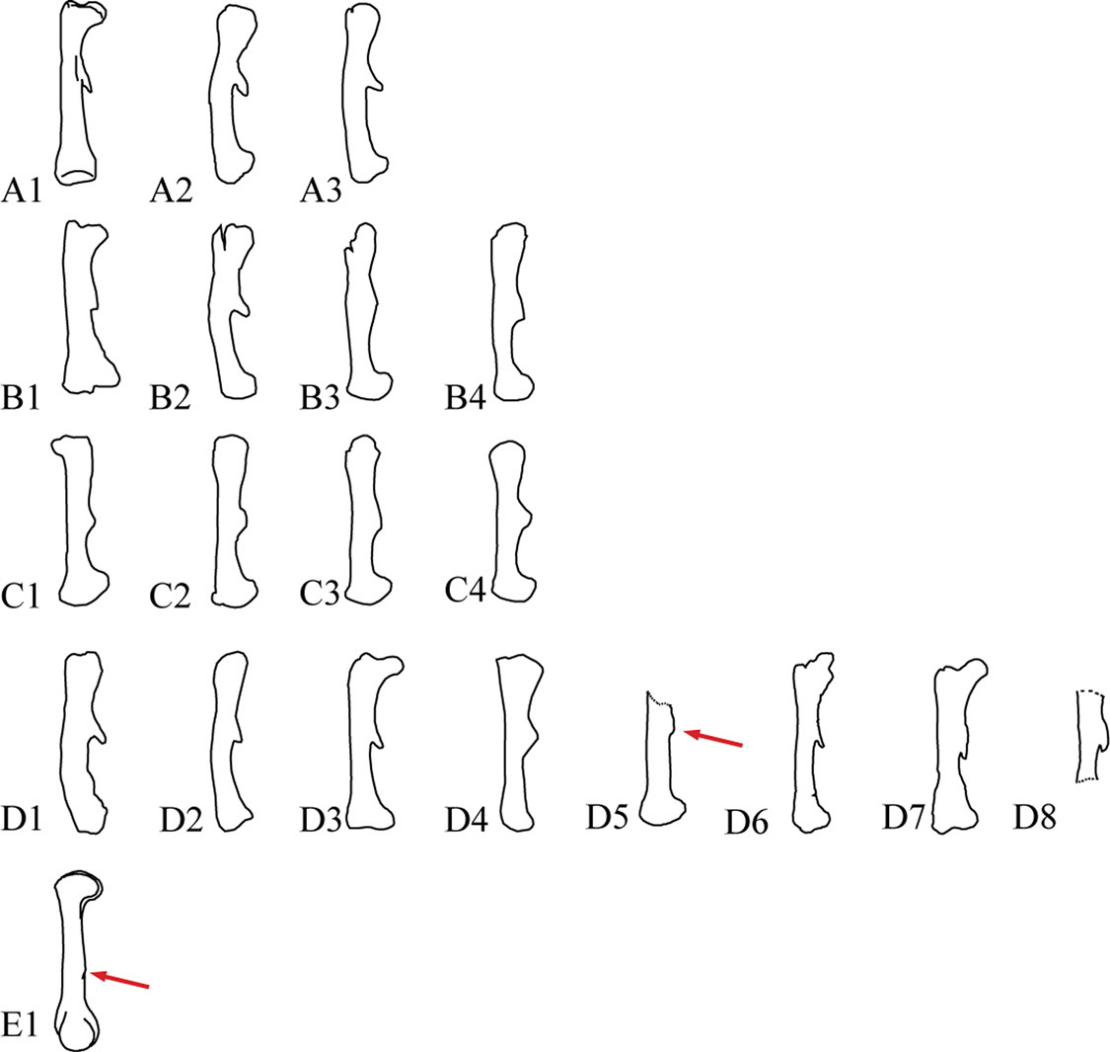
Ornithischian left femur in lateral view. All figures are just outlines, similar but not identical to the original image. All figures are for illustrative purposes only. Outlines are not to scale. A1, *Hypsilophodon foxii*, outlined from [34]; A2, *Haya griva*, based on IGM 100/2017, outlined from [33]; A3, *Jeholosaurus shangyuanensis*, based on IVPP V15939, outlined from [20]; B1, *Tenontosaurus tilleti*, outlined from [48]; B2, *Dryosaurus altus*, outlined from [49]; B3, *Probactrosaurus gobiensis*, based on PIN 2232/39-1, outlined from [68]; B4, *Iguanodon atherfieldensis*, outlined from [50]; C1, *Brachylophosaurus canadensis*, outlined from [69]; C2, *Maiasaura peeblesorum*, outlined from [69]; C3, *Hypacrosaurus stebingeri*, outlined from [69]; C4, *Corythosaurus casuarius*, outlined from [53]; D1, *Yinlong downsi*, based on IVPP V18637; D2, Psittacosaurus neimongoliensis, outlined from [55]; D3, *Auroraceratops rugosus*, outlined from [29]; D4, *Protoceratops andrewsi*, based on AMNH 6251, outlined from [70]; D5, *Leptoceratops gracilis*, based on AMNH 5205, outlined from [56]; D6, *Montanoceratops cerorhynchus*, based on AMNH 6466, outlined from [28]; D7, *Ischioceratops zhuchengensis*, based on ZCDM V0016; E1, *Triceratops horridus*, based on AMNH 4842, outlined from [59]. Arrows indicate position of fourth trochanters.

A pendant fourth trochanter occurs on the caudomedial border of the shaft, and has a length of nearly 5.5 cm (Fig 9B). This trochanter is parallelogram-shaped in medial view (Fig 10D6). Proximally, it is blade-like with a wrinkled margin, and it terminates distally in a short, triangular peak. The anteroventral margin of the distal end of the trochanter is very short and forms an angle of approximately 25° with the shaft. The fourth trochanter is closely similar in shape to that of *Montanoceratops* (Fig 10D6, [28], personal observation). The distance from the distal end of the fourth trochanter to the distal end of the femur is approximately half of the total femoral length. In many other ornithischian dinosaurs, the fourth trochanter is also pendant, but is rather different in shape (Fig 10A1–10D6). The fourth trochanter is pendant and triangular in basal ornithopods (Fig 10A1–10A2) and basal ceratopsians (Fig 10D1–10D3), although it is reduced in ceratopsids (Fig 10E1, [23]).

#### Tibia

**Fig 11:**
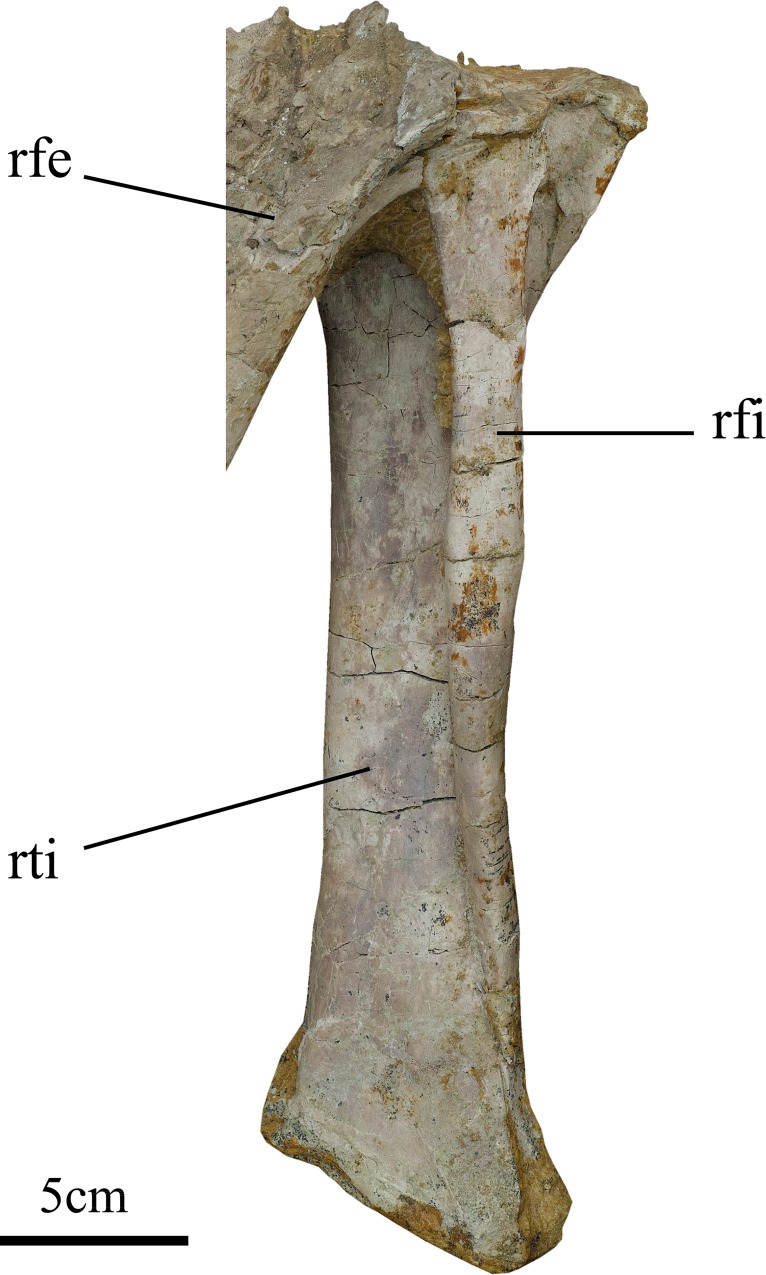
Right tibia and fibula of holotype of *Ischioceratops zhuchengensis* (ZCDM V0016). Posterolateral view. Abbreviation: rfe, right femur; rfi, right fibula; rti, right tibia.

The articulated left tibia and fibula are almost completely preserved in the new specimen (Fig 11). The tibia remains embedded in the matrix, with only the lateral side exposed. The length is approximately 32.9 cm. The ratio of tibial length to femoral length is nearly 1.13, similar to values for other derived neoceratopsians [23].

The proximal end of the tibia is partly covered by the distal end of the femur. The anterior border of the proximal end is convex, and the lateral border is strongly concave. A slim, mediolaterally compressed cnemial crest is present near the lateral border of the proximal end, and extends cranially in proximal view. The entire proximal articular surface is gently concave. Distally, the shaft forms a condyle that appears wedge-shaped, tapering distally in lateral view.

#### Fibula

The fibula is an elongate, slender, rod-like element whose distal portion articulates with the anterior side of the tibia (Fig 11). The proximal end of the fibula has a reniform outline as *Auroraceratops* [29]. The most distal part of the fibula is still buried in the matrix.

## Phylogenetic Analysis

Because of the absence of preserved craniomandibular material, and because all well-established synapomorphies of Ceratopsia pertain to the skull and mandible [28], our initial assessment of *Ischioceratops zhuchengensis* as a ceratopsian was necessarily tentative. We further investigated the systematic position of *Ischioceratops zhuchengensis* by scoring this species into a character-taxon matrix with broad coverage across Ornithischia compiled from previous studies [71–78]. A full list of characters is provided in S1 File. We also added *Koreaceratops*, *Cerasinops*, *Udanoceratops*, *Zhuchengceratops* and *Montanoceratops* to the data set, basing all codings for these taxa on the literature. The coding for character 321 of *Leptoceratops* was changed from 2 to 1, following examination of a figure of the femoral fourth trochanter by Brown [79]. *Ischioceratops zhuchengensis* could be coded for 42 of the 346 characters in the matrix.

**Fig 12:**
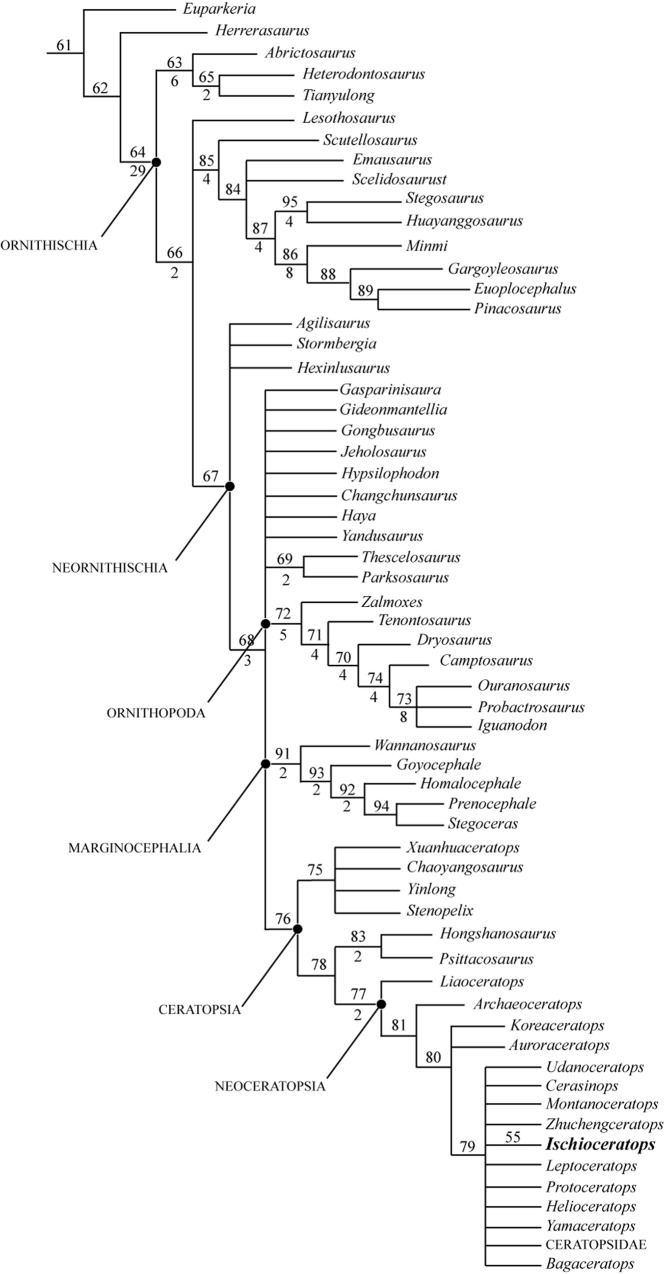
Strict consensus tree of ornithischian relationships produced by analyzing a data matrix of 61 taxa and 346 characters (see text and Supplementary Information); Values above nodes represent bootstrap proportions. Values beneath nodes indicate Bremer support. Bremer support values of +1 or less are not shown. Tree length = 1046 steps, RI = 0.707, CI = 0.385.

The matrix (Table A in S1 File) was analyzed using TNT [80], using the tree bisection reconnection algorithm, with 10,00 replicates, up to 10,000 trees saved per replication, and branches with a minimum length of 0 collapsed. All characters were unordered and treated equally. *Euparkeria capensis* [81, 82] was included in the analysis as the outgroup, and all characters were unordered. Bremer support values were also calculated, along with bootstrap support (using sampling with replacement and 1,000 replicates). 192 most parsimonious trees were recovered, each of which had a length of 1046 steps, a consistency index of 0.385 and a retention index of 0.707. This analysis confirmed that *Ischioceratops* was a basal neoceratopsian close to leptoceratopsids, protoceratopsids and Ceratopsidae (Fig 12).

In order to more effectively assess the position of *Ischioceratops* within Ceratopsia, the specimen was coded in the data matrix published by Farke et al [83], which was modified from earlier matrices [1, 30, 44, 74, 77, 84] (S2 File). We changed character (130) to the following: Femoral fourth trochanter triangular and pendant (0) or parallelogram-shaped and pendant (1) or ridge-like (2) or reduced (3). We changed character (154) to the following: Dorsal border of iliac blade vertical (0) or strongly everted (1). As a result of detailed observations from *Ischioceratops* and other basal ceratopsians, the following new character (number 155) was added to the matrix: Neural spines of middle caudal vertebrae: no longer than neural spines of anterior caudals (0) or longer than neural spines of anterior caudals (1)

**Fig 13:**
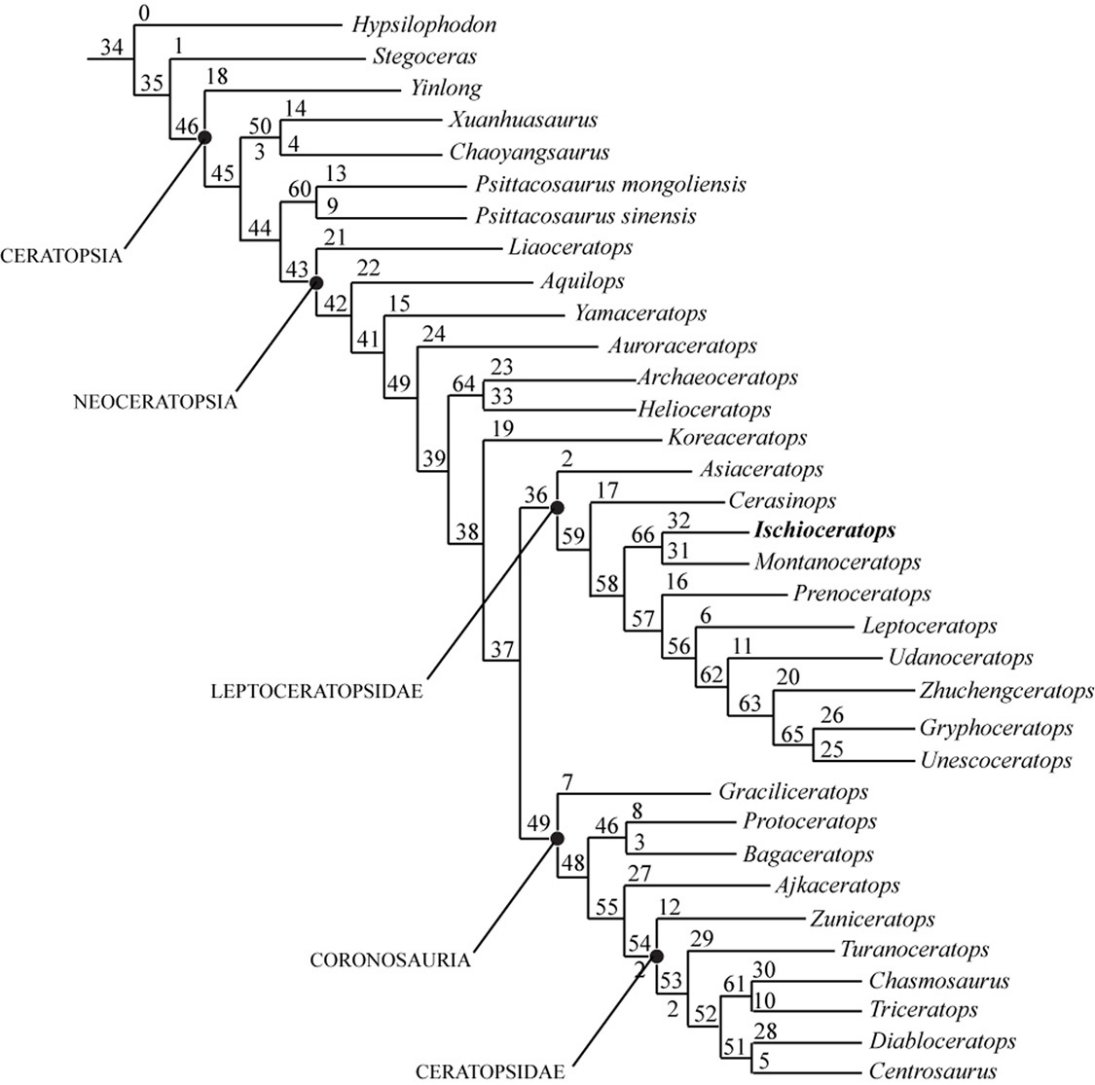
Position of *Ischioceratops zhuchengensis* among the Ceratopsia as recovered by an analysis of ceratopsian phylogenetic relationships. A single most parsimonious tree was produced by analyzing a data matrix of 34 taxa and 162 characters (see text and Supporting Information). Values above nodes represent bootstrap proportions. Values beneath nodes indicate Bremer support. Bremer support values of +1 or less are not shown. Tree length = 321 steps, CI = 0.598, RI = 0.797. Bootstrap values are shown above branches.

The matrix (Table A in S2 File) was run in TNT 1.1 [80] using the tree bisection reconnection algorithm, with 10,00 replicates, up to 10,000 trees saved per replication, and branches with a minimum length of 0 collapsed. *Hypsilophodon foxii* [85] was included as an outgroup taxon, and all characters were unordered. Bremer support values were also calculated, along with bootstrap support (using sampling with replacement and 10,000 replicates). All characters were unordered and equally weighted. The analysis resulted in only one most parsimonious tree, with a length of 315 steps, a consistency index of 0.603 and a retention index of 0.801. *Ischioceratops* was recovered as a derived leptoceratopsid (Fig 13), and as the sister taxon to *Montanoceratops*. The Bremer support value showed that it can be moved as sister taxon to *Zhuchengceratops* by only one step.

## Discussion

Well-established synapomorphies of Ceratopsia are mainly craniomandibular, and include the presence of a rostral bone, prominent jugal horns, a vaulted premaxillary palate, and a predentary with a broad base that supports the dentary symphysis [27]. Unfortunately, few postcranial synapomorphies have been identified [30]. However, the preserved postcranial skeletal elements that are available for *Ischioceratops* display features that preclude referral to any major ornithischian clade outside Ceratopsia, or identification of *Ischioceratops* as a ceratopsid. The nine sacral vertebrae exclude *Ischioceratops* from identification as a basal ornithopod or ankylosaurid and the lateral outline of ilium without a lateral everted shelf on the dorsal edge excludes *Ischioceratops* from iguanodontians, hadrosaurids, and ceratopsids. The increasing elongation of more posteriorly situated neural spines in the proximal half of the tail is similar to the condition in several basal ceratopsian dinosaurs, and a pendant, parallelogram-shaped fourth trochanter on the femur is similar to that in *Montanoceratops*.

The principal diagnostic feature of *Ischioceratops* is the fenestrated midshaft expansion of the ischial shaft. This highly unusual feature renders the ischium unlike that of any other dinosaur. While this unusual morphology prompts the question of whether it could be the result of pathology, several factors argue against this interpretation. Firstly, the expansion and opening occur symmetrically on both ischia, and both ischia bear a medial groove extending distally from the midshaft expansion. Furthermore the openings in the midshaft expansion appear to be true fenestrae with finished edges rather than the blind recesses sometimes observed in connection with tendon avulsions [86] or pus canals [87]. We therefore assume that the ischial morphology observed in this specimen represents the normal condition in *Ischioceratops*, rather than a pathological anomaly.

Although it is tempting to homologize the ischial expansions of *Ischioceratops* with the obturator processes of more basal ornithopods/cerapodans, this is problematic for both topological and phylogenetic reasons. In basal ornithischians that have an obturator process, such as *Hypsilophodon* (Fig 8A3, [34] and *Tenontosaurus* (FMNH PR 2173), this structure is restricted to the ventral border of the ischial shaft and is located well proximal to the midpoint of the shaft. By contrast, the expansion in *Ischioceratops* arises from the ventrolateral edge of the shaft and located distal to the midlength of the ischium. Furthermore, no other marginocephalian taxa exhibit obturator processes, and both of our phylogenetic analyses support a relatively derived position for *Ischioceratops* within Neoceratopsia. Thus, the ischial shaft expansion and fenestra are best viewed as a neomorphic character that is currently only known in *Ischioceratops* and unexpectedly increases the known morphological disparity of the otherwise rather conservative leptoceratopsid pelvis.

Another unique aspect of the *Ischioceratops* ischium is the knob-like distal expansion. This feature absent in other ornithischians, although a differently-shaped terminal expansion of the ischium is present in several non-hadrosauroid iguanodontians (Fig 8B3–8B5) [39, 52], some basal hadrosaurines (Fig 8C2–8C4) [52] and some basal neoceratopsians (e.g. *Auroraceratops*, Fig 8E4, *Protoceratops*, Fig 8E5). In these species, the foot-like structure expands ventrally at a 90° angle from the main shaft [39], whereas in *Ischioceratops* the distal end of the ischium is expanded both dorsally and ventrally. The ischial shaft is unexpanded in most other neoceratopsian taxa in which it is known (Fig 8E2–8F4), although in *Yinlong* the middle portion of the ischium is ventrally expanded in lateral view. As with the midshaft expansion, the knob-like distal swelling appears to be an autapomorphy of *Ischioceratops* rather than a retained primitive feature.

In 2008, *Zhuchengceratops inexpectus* and *Sinoceratops zhuchengensis* were excavated from the bone-beds of the Upper Cretaceous Wangshi Group of Zhucheng, Shandong Province, at the Kugou and Zangjiazhuang localities respectively [11]. Numerical phylogenetic analyses positioned *Zhuchengceratops* as a derived leptoceratopsid within a clade also containing *Montanoceratops*, *Udanoceratops*, and *Leptoceratops* [10].

The holotype of *Ischioceratops* was found at approximately the same stratigraphic level within the Kugou quarry as the holotype specimen of *Zhuchengceratops*, raising the question of whether the two specimens may be conspecific. Unfortunately, there are no overlapping skeletal elements between the two specimens. It depends on the recovery of overlapping material and future discoveries. *Zhuchengceratops* was recovered by our phylogenetic analysis has a close relationship with *Ichioceratops* in Leptoceratopsidae. Therefore, we provisionally consider *Ischioceratops* and *Zhuchengceratops* to be distinct taxa, although we acknowledge that future discoveries might reveal them to be synonymous.

## Supporting Information

S1 File Character list for analysis of ornithischian phylogenetic relationships. Table A. Codings for S1 phylogenetic analysis, in TNT format.
(DOC)

S2 File Character list for analysis of ceratopsian phylogenetic relationships. Table A. Codings for S3 phylogenetic analysis, in TNT format
(DOC)

## Abbreviations

AMNH: American Museum of Natural History, New York, USA
FMNH: Field Museum of Natural History, Chicago, USA
HNM: Humboldt Museum fur Naturkunde, Berlin, Germany
IVPP: Institute of Vertebrate Palaeontology and Paleoanthropology, Beijing, China
MOR: Museum of the Rockies, Bozeman, USA
NMC: Canadian Museum of Nature, Ottawa, Canada
ROM: Royal Ontario Museum, Toronto, Canada
TMM: Texas Memorial Museum, Austin, USA
YPM: Peabody Museum of Natural History, Yale University, New Haven, USA
ZCDM: Zhucheng Dinosaur Museum, Zhucheng, China
ZDM: Zigong Dinosaur Museum, Zigong, China
