# Supplementary material for: A New Leptoceratopsid (Ornithischia, Ceratopsia) with a Unique Ischium from the Upper Cretaceous of Shandong Province, China
Source: PLoS One. 2015 Dec 23;10(12):e0144148. doi: 10.1371/journal.pone.0144148 (PMC4689537; doi:10.1371/journal.pone.0144148)
Supplement: S1 File — Table A. Codings for S1 phylogenetic analysis, in TNT format. (DOC) [file pone.0144148.s001.doc]

### Supporting information.

**S1 File. Character list for analysis of ornithischian phylogenetic relationships**. “#” indicates characters whose codings have been modified.

1 Head shape in dorsal view: elongate, ovoid (0), triangular, truncated (1); or wide over jugals (2). [Maryańska and Osmólska, 1985, (35); Sereno, 1986; Xu et al., 2002, (2); Liu, 2004, (1)]

2 Skull proportions, preorbital skull length relative to basal skull lenth: less than 40% of basal skull length (0); more than 40% of basal skull length (1). [Weishampel et al., 2003, (1); Butler et al., 2008, (1)] additive character

3 Skull length (rostral-quadrate): 15% or less of body length (0); 1, 20–30% of body length (1). [Butler et al., 2008, (2)]

4 Neomorphic rostral bone, anterior to premaxilla: absent (0); 1, present (1). [Butler et al., 2008, (3)]

5 Rostral bone, anteriorly keeled and ventrally pointed: absent (0); present (1). [Butler et al., 2008, (4)]

6 Rostral ventral (buccal) process: absent (0), or present (1). [Makovicky and Norell, 2006, (7)]

7 Premaxilla, posterolateral process, length: does not contact lacrimal (0); contact the lacrimal, excludes maxilla–nasal contact (1). [Butler et al., 2008, (7)]

8 Position of the ventral (oral) margin of the premaxilla: level with the maxillary tooth row (0); deflected ventral to maxillary tooth row (1). [Butler et al., 2008, (9)]

9 Premaxillary foramen: absent (0); present (1). [Butler et al., 2008, (10)]

10 Overlap of the dorsal process of the premaxilla onto the nasal: present (0); absent (1). [Butler et al., 2008, (12)]

11 Fossa-like depression positioned on the premaxilla–maxilla boundary: absent (0); present (1). [Butler et al., 2008, (13)]

12 Arched “subnarial gap” between the premaxilla and maxilla: absent (0); present (1). [Butler et al., 2008, (15): modified]

13 Premaxilla-prefontal contact: absent (0) or present (1). [Xu et al., 2002(102) ; 2006, (23)]

14 Oral margin of the premaxilla: narial portion of the body of the premaxilla slopes steeply from the external naris to the oral margin (0); ventral premaxilla flares laterally to form a partial floor of the narial fossa (1). [Butler et al., 2008, (8)]

15 Premaxilla, edentulous anterior region: absent, first premaxillary tooth is positioned adjacent to the symphysis (0); present, first premaxillary tooth is inset the width of one or more crowns (1). [Butler et al., 2008, (6)]

16 Premaxillary palate: strongly arched, forming a deep, concave palate (0); horizontal or only gently arched (1). [Butler et al., 2008, (11)]

17 External naris, position: confined to the area immedially above the oral margion of the premaxilla (0); extending posteriorly so as to lie above the maxilla (1). [Weishampel et al., 2003: 2]

18 External nares, position of the ventral margin: below the ventral margin of the orbits (0); above the ventral margin of the orbits (1). [Xu et al., 2006, (8); Butler et al., 2008, (17)]

19 Ventral border of external nares: significantly below (0); about the level (1); or significantly above (2) that of the infratemporal fenestra. [Xu et al., 2002, (100); Liu, 2004 (28)] additive character

20 Relative height of snout at nares to orbital region: low, height at mid naris level about 35% of that at the mid orbit level (0); deep, about or more than 60%(1)[Xu et al., 2006: 7]

21 Narial fossa, maximum length: shorter than (0), subequal to (1), or twice as long as (2) maximum anteroposterior diameter of the orbit. [Sereno, 1986; Weishampel and Heinrich, 1992, (2); Sereno, 1999, 3(14); Liu, 2004, (26)]

22 Deep elliptic fossa present along sutural line of the nasals: absent (0); present (1). [Butler et al., 2008, (19) ]

23 Nasal horn: absent (0); present (1). [Makovicky and Norell, 2006, 18]

24 Anterior end of the nasal: above (0); or below and far (1) rostral to the external naris. [Makovicky and Norell, 2006, 14]

25 Nasal septum dividing narial passage: absent (0); present (1). [Sereno, 1986; 1999, 2(57); Hill et al., 2003(14); Liu, 2004 (29)]

26 Edentulous maxillary anterior margin, length: 0 (0); about 2 (1), or 4~5 (2), tooth spaces. [Sereno, 1999, 4(57); Liu, 2004, (160)]additive character

27 Antorbital fossa (external antorbital fenestra): absent (0); present (1). [Butler et al., 2008: 21]

28 External antorbital fenestra, shape: triangular (0); oval or circular (1). [Butler et al., 2008, (22)]

29 Additional opening(s) anteriorly within the antorbital fossa: absent (0); present (1). [Butler et al., 2008, (23) ]

30 Antorbital fossa margins: all sharply defined (0); or some margin poorly delineated (1). [Xu et al., 2006, (53)]

31 External antorbital fenestra, position: entirely anterior to orbit (0), or fenestra below orbit (1). [Xu et al., 2006, (77)]

32 Maxilla, prominent anterolateral boss articulates with the medial premaxilla: absent (0); present (1). [Butler et al., 2008, (24) ]

33 Maxilla, accessory anterior process: absent (0); present (1). [Weishampel et al., 2003; Butler et al., 2008, (25)]

34 Maxilla, buccal emargination: absent (0); present (1). [Butler et al., 2008, (26)]

35 Eminence on the rim of the buccal emargination of the maxilla near the junction with the jugal: absent (0); present (1). [Butler et al., 2008, (27)]

36 Slot in maxilla for lacrimal: absent (0); present (1). [Butler et al., 2008, (28)]

37 Maxilla-jugal suture. Scarf joint (0), ‘finger-in-recess’ joint (1).[Norman, 2002, (15); Liu, 2004, (22)]

38 Accessory ossification (s) in the orbit (palpebral/supraorbital): absent (0); present (1). [Butler et al., 2008, (29) ]

39 Palpebral/supraorbital: free, projects into orbit from contact with lacrimal/prefrontal (0); incorporated into orbital margin (1). [Butler et al., 2008, (30) ]

40 Palpebral length: more than 80% of the length of the orbit (0); shortening, less than 70% of the length of the orbit (1). [Weishampel and Heinrich, 1992, (9); Liu, 2004, (39)]

41 Palpebral, shape in dorsal view: rod-shaped (0); platelike with wide base (1). [Butler et al., 2008, (31) ]

42 Palpebral/supraorbital, number: one (0); two (1); three (2). [Butler et al., 2008, (32) ]

43 Exclusion of the jugal from the posteroventral margin of the external antorbital fenestra by lacrimal–maxilla contact: absent (0); present (1). [Butler et al., 2008, (34)]

44 Jugal contribution to antorbital fossa: small (0) or significant (1). [Xu et al., 2006, (79)]

45 Anterior ramus of jugal, proportions: deeper than wide, but not as deep as the posterior ramus of the jugal (0); wider than deep (1); deeper than the posterior ramus of the jugal (2). [Butler et al., 2008, (35) ]

46 Jugal anterior end, shape: tapered (0); expanded dorsoventrally (1). [Sereno, 1986; 1999, 3(86); Norman, 2002, (14) part; Kobayashi and Azuma, 2003, (5); Liu, 2004, (40)]

47 Jugal (or jugal-epijugal) ridge dividing the lateral surface of the jugal into two planes: absent (0); present (1). [Butler et al., 2008, (38) ]

48 Jugal boss: absent (0); present (1). [Butler et al., 2008, (40)]

49 Node-like ornamentation on jugal, mostly on, or ventral to the jugal–postorbital bar: absent (0); present (1). [Butler et al., 2008, (41)]

50 Ventral edge of the jugal: Straight or shallow curve (0), strongly angular (1). [Norman, 2002, (16); Liu, 2004, (45)]

51 Jugal-postorbital bar, width broader than laterotemporal fenestra: absent (0); present (1). [Butler et al., 2008, (42)]

52 Jugal-squamosal contacts above infratemporal fenestra: absent (0); present (1). [Butler et al., 2008, (45)]

53 Jugal posterior process, shape: tapering (0), forked (1), weakly expanded (2); strongly expanded and bluntly truncated (3). [Benton, 1999, (4); Sereno, 1999, 1(64); Liu, 2004, (41)]

54 Jugal, posterior ramus: forms anterior and ventral margin of infratemporal fenestra (0); forms part of posterior margin, expands towards squamosal (1). [Butler et al., 2008, (47)]

55 Jugal-quadratojugal contact: overlapping (0); tongue and-groove (1). [Butler et al., 2008, (48)]

56 Jugal horn: absent (0); present (1). [Makovicky and Norell, 2006:27]

57 Epijugal: absent (1); present (0). [Sereno, 2000: 31]

58 Postorbital surface: smooth (0) or with a prominent vertical ridge (1). [Xu et al., 2006, (86)]

59 Postorbital, orbital margin: relatively smooth curve (0); prominent and distinct projection into orbit (1). [Butler et al., 2008, (49) ]

60 Squamosal process of postorbital relative to the jugal process: much shorter (0) or subequal or longer than (1). [Xu et al., 2006, (83)]

61 Postorbital: T-shaped (0); triangular and plate-like (1). [Butler et al., 2008, (50) ]

62 Postorbital–parietal contact: absent, or very narrow (0); broad (1). [Butler et al., 2008, (51) ]

63 Postorbital and supratemporal bars, maximum width: narrow, bar-shaped (0); broad, strap-shaped (1); very broad, plate-shaped (2).[Sereno, 1986; 1999, 4(9), 4(63); Liu, 2004, (56)]

64 Quadratojugal shape: “L” shaped (0); “T” shaped (1). [Sereno, 2012, 20]

65 Quadratojugal foramen: absent (0); present (1). [Weishampel et al., 2003, (17) ]

66 Quadratojugal, extent of jugal overlap: anterior edge (0); majority of lateral surface (1). [Sereno, 1999, 3(62); Liu, 2004, (86)]

67 Contact between dorsal process of quadratojugal and descending process of the squamosal: present (0); absent (1). [Butler et al., 2008, (52)]

68 Quadratojugal, orientation: faces laterally (0); faces posterolaterally (1). [Butler et al., 2008, (55)]

69 Quadratojugal, transverse width: mediolaterally flattened (0); transversely expanded and triangular in coronal section (1). [Sereno, 1986; 1999, 2(56); Liu, 2004, (87); Butler et al., 2008, (56)]

70 Quadrate lateral ramus: present (0); absent (1). [Butler et al., 2008, (58) ]

71 Quadrate shaft, anteroposterior width relative to height: broad, more than 1/4 (0); or narrow less than 1/5 (1). [Sereno, 1999, 4(66); Liu, 2004, (75)]

72 Quadrate shaft: anteriorly convex in lateral view (0); straight (1). [Xu et al., 2002, (31); Liu, 2004, (74)]

73 Quadrate, free portion of shaft: 10% or less (0), or 30% or more (1), of quadrate height. [Sereno, 1999, 3(24); Liu, 2004, (77)]

74 Quadrate condyle, inclination of articular surface (posterior view): horizontal (0); ventromedially inclined at approximately 45 degrees (1); ventrolaterally inclined (2). [Sereno, 1986; 1999, 2(14); Liu, 2004, (79)]

75 Quadrate (paraquadratic) foramen: present (0); absent (1). [Liu, 2004, (80)]

76 A paraquadratic foramen restricted within the posterior margin of quadrate absent (0) or present (1). [Xu et al, 2006:59]

77 Dorsoventrally narrow pterygoid ramus of the quadrate: absent (0); present (1). [Sereno, 1986; 1999, 2(60); Hill et al., 2003, (23); Liu, 2004, (83)]

78 Prominent oval fossa on pterygoid ramus of quadrate: absent (0); present (1). [Butler et al., 2008, (57)]

79 Quadrate mandibular articulation: quadrate condyles subequal in size (0); medial condyle is larger than lateral condyle (1); lateral condyle is larger than medial (2). [Butler et al., 2008, (63)]

80 Frontal excluded from the orbit: absent (0); present (1). [Liu, 2004, (53)]

81 Frontal participation in supratemporal fenestra: absent (0); present (1). [Sereno, 1999, 1(2); Liu, 2004, (54)]

82 Paired frontals: short and broad (0); narrow and elongate (more than twice as long as wide) (1). [Butler et al., 2008, (64)]

83 Postfrontal: present (0); absent (1). [Benton, 1999, (5); Sereno, 1999, 1(1); Liu, 2004, (64)]

84 Supratemporal fenestra: present (0); absent (1). [Butler et al., 2008, (65)]

85 Supratemporal fenestra, anteroposteriorly elongated: absent, fenestrae are subcircular to oval in shape (0); present (1). [Butler et al., 2008, (66)]

86 Supratemporal fenestra length relative to the basal skull length: short, less than 20% basal skull length (0); or elongated, more than 25% basal skull length (1). [Xu et al., 2006, (58)]

87 Infratemporal fenestra: open (0); closed (1).[ Hill et al., 2003; (35); Liu, 2004, (60)]

88 Infratemporal fenestra size: small, much smaller than the orbit (0) or large, subequal or larger than the orbit (1). [Xu et al., 2006, (87)]

89 Parietals: paired (0); fused (1). [Gauthier, 1986; Liu, 2004 (65)]

90 Parietal, location of posterior margin relative to squamosal: anterior to (0) or level with or posterior to (1) that of squamosal. [Xu et al., 2006, (60)]

91 Parietal sagittal crest: narrow and sharply defined (0) or broad, essentially absent (1). [Xu et al., 2006, (102)]

92 Parietal fenestration: absent (0) or present (1). [Xu et al., 2006, (71)]

93 Parietal width: subequal to (0); or much wider than (1) the dorsal skull roof. [Sereno, 2000, 53]

94 Parietal septum, form: narrow and smooth (0); broad and rugose (1). [Butler et al., 2008, (67)]

95 Frontal and parietal dorsoventral thickness: thin (0); thick (1). [Butler et al., 2008, (74)]

96 Frontoparietal doming: absent (0); present (1). [Sereno 2000]

97 Parietosquamosal shelf: absent (0) or present (1). [Xu et al., 2006, (45)]

98 Parietosquamosal shelf, extended posteriorly as distinct frill: absent (0), present(1).[Butler et al., 2008:69]

99 Composition of the posterior margin of the parietosquamosal shelf: parietal contributes only a small portion to the posterior margin (0); parietal makes up at least 50% of the posterior margin (1). [Butler et al., 2008, (70)]

100 Posterior edge of squamosal: angled anteromedially (0); nearly on one line (1); angled posteromedially, squamosal contributing to frill margin (2). [Maryańska and Osmólska, 1985, (33); Xu et al., 2002, (115); Liu, 2004, (71)]

101 Squamosal-quadrate articulation position: close to the main body of the squamosal (0), away from the main body of the squamosal, on a distinct, robust ventral process (1). [Xu et al., 2006, (100)]

102 Anterior process of the squamosal articulate to the posterior process of the postorbital: simple, scarf joint (0); deeply bifurcate (1). [Makovicky and Norell, 2006, (37)]

103 Postorbital–squamosal tubercle row: absent (0); present (1). [Butler et al., 2008, (72)]

104 Enlarged tubercle row on the posterior squamosal: absent (0); present (1). [Butler et al., 2008, (73)]

105 Paroccipital process, proportions: short and deep (height ≥ 1/2 length) (0); elongate and narrow (1). [Butler et al., 2008, (76) ]

106 Paroccipital process, shape of ventral tip: subtriangular (0); rod-shaped (1). [Sereno, 1999, 3(32); Liu, 2004, (102)]

107 Supraoccipital, contribution to dorsal margin of foramen magnum: 0, forms entire dorsal margin of foramen magnum; 1, exoccipital with medial process that restricts the contribution of the supraoccipital. [Butler et al., 2008, (78) ]

108 Occiput, the ratio of maximum width to maximum height: >1.1 (0), or 0.9~1.1 (1), <0.9 (2). [Sereno, 1986; 1999, 2(52); Hill et al., 2003, (28); Liu, 2004, (90)] additive characters

109 Basioccipital, a longitudinal ridge below condyle: present (0); very weak or absent (1) [Xu et al., 2006, (95)] 保留修改

110 Basisphenoid: longer than or subequal in length to, basioccipital (0); shorter than basioccipital (1). [Butler et al., 2008, (80)]

111 Prootic–basisphenoid plate: absent (0); present (1). [Butler et al., 2008, (81)]

112 Basal tubera, shape: knob-shaped (0); plate-shaped (1). [Butler et al., 2008, (82)]

113 Basipterygoid processes, orientation: anteroventral (0); ventral (1); posteroventral (2). [Butler et al., 2008, (83)]

114 Basipterygoid process articular facet for the pterygoid small and oval (0) or large and elliptical (1) in outline. [Xu et al., 2006, (96)]

115 Basipterygoid process articular facet orientation: mainly directed anteriorly (0) or laterally (1). [Xu et al., 2006, (97)]

116 Pterygoid prominent posterior process: absent (0); present (1).. [Xu et al., 2002, (106)]

117 Pterygoid-ectopterygoid articular relation: ectopterygoid ventral (0); ectopterygoid dorsal (1). [Sereno, 1999, 1(3); Liu, 2004 (113)]

118 Pterygoid–maxilla contact at posterior end of tooth row: absent (0); present (1). [Butler et al., 2008, (87)]

119 Pterygoquadrate rami, posterior projection of ventral margin: weak (0); pronounced (1). [Butler et al., 2008, (88) ]

120 Pterygoid, vomeral process: anteriorly oriented (0) or dorsally oriented (1). [Xu et al., 2006, (62) ]

121 The anterior margin of choana, position on palate: anterior to maxillary tooth row (0); level with the anteriormost maxillary tooth (1), posterior to the anteriormost maxillary tooth (2). [Liu, 2004 (118)]

122 Premaxilla–vomeral contact: present (0); absent, excluded by midline contact between maxillae (1). [Butler et al., 2008, (84) ]

123 Dorsoventrally deep (deeper than 50% of snout depth) median palatal keel formed of the vomers, pterygoids and palatines: absent (0); present (1). [Butler et al., 2008, (85)]

124 Extension of the vomerine septum: incomplete (0); extending to palatal shelves (1); extending to skull roof (2). [Hill et al., 2003, (18); Liu, 2004, (115)]

125 Secondary palate: incomplete or absent (0); complete (1). [Hill et al., 2003, (20); Liu, 2004, (116)]

126 Cortical remodeling of surface of skull dermal bone: absent (0); present (1). [Butler et al., 2008, (89)]

127 Obliteration of cranial sutures in adults, involving fusion and dermal sculpturing of the outer surface of most of the dermal skull roof: absent (0); present (1). [Sereno, 1986; 1999, 2(63); Hill et al., 2003; (36); Liu, 2004, (119)]

128 Two pairs of dermal ossifications bordering the external nares: absent (0); present (1) [Sereno, 1986; 1999, 2(92); Hill et al., 2003, (44); Liu, 2004, (120)]

129 Secondary dermal ossification, projecting ventrolaterally from the quadratojugal region: absent (0); present and rounded (1); present and wedge-shaped (2). [Hill et al., 2003, (41); Liu, 2004, (121)]

130 Secondary dermal ossification, projecting caudolaterally from the squamosal region: absent (0); present as weakly developed pyramid (1); present as prominent, wedge-shaped or pyramidal structure (2). [Hill et al., 2003, (42); Liu, 2004, (122)]

131 Posteroventral dermal plate, position: posterior (0), or posteroventral (1), to orbit. [Sereno, 1999, 2(105); Liu, 2004, (123)]

132 Predentary: absent (0); 1, present (1). [Butler et al., 2008, (90) ]

133 Predentary size: short, posterior premaxillary teeth oppose anterior dentary teeth (0); roughly equal in length to the premaxilla, premaxillary teeth only oppose predentary (1). [Butler et al., 2008, (91) ]

134 Predentary, rostral end in dorsal view: rounded (0); pointed (1). [Butler et al., 2008, (92) ]

135 Predentary, oral margin: relatively smooth (0); denticulate (1). [Butler et al., 2008, (93) ]

136 Tip of predentary in lateral view: does not project above the main body of predentary (0); strongly upturned relative to main body of predentary (1). [Butler et al., 2008, (94)]

137 Predentary, ventral process: present, well-developed (0); very reduced or absent (1). [Butler et al., 2008, (96)]

138 Predentary, ventral process: single (0); bifurcated (1). [Butler et al., 2008, (95)]

139 Predentary ventral process, width of base: less (0), or equals to or more (1), than half the maximum transverse width of the predentary.[Sereno, 2000: Ceratopsia 7]

140 Predentary length of lateral process relative to the ventral process: short (0) or long (1). [Xu et al., 2006, (93)]

141 Dentary symphysis: V-shaped (0); spout shaped (1). [Butler et al., 2008, (97)]

142 Dentary tooth one, position relative to the predentary: adjacent (0); separated by a short diastema (1); separated by a long diastema (2). [Sereno, 1986; 1999, 3(68); Xu et al., 2002, (55); Norman, 2002, (21); Kobayashi and Azuma, 2003, (11); Liu, 2004, (162)]additive characters

143 Diastema distal to dentary caniniform tooth: absent (0); present (1). [Sereno, 2012: 5]

144 #Dentary tooth row (and edentulous anterior portion) in lateral view: straight (0); anterior end downturned (1); anterior end upturned. [Butler et al., 2008, (98)]

145 Dentary tooth row (and anterior edentulous margin), form (lateral view): straight (0); sinuous (1). [Sereno, 1986; 1999, 2(4); Liu, 2004, (167)]

146 Dorsal and ventral margins of the dentary: 0, converge anteriorly; 1, subparallel. [Butler et al., 2008, (99)]

147 Ventral flange on dentary: 0, absent; 1, present. [Butler et al., 2008, (100)]

148 Dentary ventral margin, form: straight (0); strongly curved (1). [Sereno, 2000,47]

149 Alveolar foramina (‘special foramina’) medial to maxillary/dentary tooth rows: present (0); absent (1). [Butler et al., 2008, (126)]

150 Coronoid process: absent or weak, posterodorsally oblique, depth of mandible at coronoid is less than 140% depth of mandible beneath tooth row (0); well-developed, distinctly elevated, depth of mandible at coronoid is more than 180% depth of mandible beneath tooth row (1). [Butler et al., 2008, (101)]

151 Coronoid process, shape: posterodorsally oblique, short coronoid process on the dentary (0); distinctly elevated process (1). [Weishampel et al., 2003: 22]

152 Anterodorsal margin of coronoid process formed by posterodorsal process of dentary: absent (0); present (1). [Butler et al., 2008, (102)]

153 Coronoid process position: close to main axis of dentary and posterior to tooth row (0) or set lateral to tooth row, and end of tooth row covered by anterior part of coronoid process (1) or tooth row level with posterior edge of coronoid process (2). [Norman, 1990; Sereno, 1999, 3(69), 4(61); Xu et al., 2002, (59); Liu, 2004, (141)] additive character

154 Postdentary bones, location: anterior (0), or posterior (1), to midline of coronoid process. [Sereno, 1986; 1999, 3(49); Kobayashi and Azuma, 2003, (13); Liu, 2004, (142)]

155 Dentary, sub-buccal portion: shallower than postdentary region (0); as deep as postdentary region (1). [Xu et al., 2006, (64)]

156 External mandibular fenestra, situated on dentary surangular-angular boundary: present (0); absent (1). [Butler et al., 2008, (104) ]

157 External mandibular fenestra, length: more than 50% maximum depth of the lower jaw (0) small or absent(1). [Gauthier 1986; Sereno, 1986; 1999, 3(8); Hill et al., 2003, (46); Liu, 2004, (143)]

158 Small fenestra positioned dorsally on the surangulardentary joint: absent (0); present.(1). [Butler et al., 2008, (105) ]

159 Surangular lateral surface in dorsal view: flat to weakly convex (0) or with pronounced laterally convex curvature (1). [Xu et al. 2006, (32)]

160 #Surangular length: less than (0) or more than (1) half of the mandible length. [Xu et al., 2006, (65)]

161 Ridge or process on lateral surface of surangular, anterior to jaw suture: absent (0); present, strong anteroposteriorly extended ridge (1); present, dorsally directed finger-like process (2). [Butler et al., 2008, (106), Han et al., 2012(106)]

162 Retroarticular process: elongate (0); rudimentary or absent (1). [Butler et al., 2008, (107)]

163 Articular, medial expansion of the glenoid to form a semilunar surface: absent (0); present (1).[Zhao and Xu , 2006

164 Node-like ornamentation of the dentary and angular: absent (0); present (1). [Butler et al., 2008, (108)]

165 Level of jaw joint: level with tooth row, or weakly depressed ventrally (0); strongly depressed ventrally, more than 40% of the height of the quadrate is below the level of the maxilla (1). [Butler et al., 2008, (109)]

166 Mandibular osteoderm: absent (0); present (1).. [Butler et al., 2008, (110)]

167 Angular tubercle row: absent (0); present (1). [Sereno, 1986; 1999, 4(15); Liu, 2004, (148)]

168 Premaxillary teeth: present (0); absent, premaxilla edentulous (1). [Butler et al., 2008, (111)]

169 Premaxillary teeth, number: above or equals to six (0); five (1); four (2); three (3); two (4); one (5). [Butler et al., 2008, (112)] additive character

170 Premaxillary teeth, size: equivalent to (0) or much larger than (1) maxillary teeth. [(Xu et al., 2006, (73)]

171 Premaxillary teeth, crown expanded above root: crown is unexpanded mesiodistally above root, no distinction between root and crown is observable (0); crown is at least moderately expanded above root (1). [Butler et al., 2008, (113)]

172 Premaxillary teeth increase in size posteriorly: absent, all premaxillary teeth subequal in size (0); present, posterior premaxillary teeth are significantly larger in size than anterior teeth (1); middle premaxillary teeth are the largest (2). [Butler et al., 2008, (114)] 保留

173 Maxillary and dentary teeth, number in tooth row: less (0), or more (1), than 20. [Sereno 1999, 3(50); Liu, 2004, (159)]

174 Maxillary and dentary crowns, shape: apicobasally tall and blade-like (0); apicobasally short and subtriangular (1); diamond-shaped (2). [Butler et al., 2008, (115)]

175 Maxillary tooth crown, shape: triangular and pointed (0), chisel-shaped with parallel sides (1) or ovate (2) in lateral view.（Xu et al., 2006: 75）

176 Apicobasally extending ridges on maxillary/dentary teeth: absent (0); present (1). [Butler et al., 2008, (118)]

177 Apicobasally extending ridges on lingual/labial surfaces of maxillary/dentary crowns confluent with marginal denticles: absent (0); present (1). [Butler et al., 2008, (119)]

178 Prominent primary ridge on labial side of maxillary teeth: absent (0); present (1). [Butler et al., 2008, (120)]

179 Prominent primary ridge on lingual side of dentary teeth: absent (0); present (1). [Butler et al., 2008, (121)]

180 Position of maxillary/dentary primary ridge: centre of the crown surface, giving the crown a relatively symmetrical shape in lingual/labial view (0); offset, giving crown asymmetrical appearance (1). [Butler et al., 2008, (122)]

181 Maxillary primary ridge: less (0), or more (1), prominent than dentary primary ridge. [Sereno, 1986; 1999, 3(27); Liu, 2004, (172)]

182 Maxillary teeth anterior and posterior ridges: weak (0); prominent (1).(Xu et al., 2006: 76)

183 At least moderately developed labiolingual expansion of crown (‘cingulum’) on maxillary/dentary teeth: present (0); absent (1). [Butler et al., 2008, (123)]

184 Heterodont dentary dentition: no substantial heterodonty is present in dentary dentition (0); single, enlarged, caniform anterior dentary tooth, crown is not mesiodistally expanded above root (1); anterior dentary teeth are strongly recurved and caniform, but have crowns expanded mesiodistally above their roots and are not enlarged relative to other dentary teeth (2). [Butler et al., 2008, (124)]

185 Peg-like tooth located anteriorly within dentary, lacks denticles, strongly reduced in size: absent (0); present (1). [Butler et al., 2008, (125)]

186 Recurvature in maxillary and dentary teeth: present (0); absent.(1). [Butler et al., 2008, (127)]

187 Overlap of adjacent crowns in maxillary and dentary teeth: absent (0); present (1). [Butler et al., 2008, (128)]

188 Crown is mesiodistally expanded above root in cheek teeth: absent (0); present (1). [Butler et al., 2008, (129)]

189 Position of maximum apicobasal crown height in dentary/maxillary tooth rows: anterior portion of tooth row (0); central portion of tooth rows (1); posterior portion of tooth row (2). [Butler et al., 2008, (130)]

190 Maxillary/dentary crown, height: subequal to (0), or 1.5 times (1), maximum crown width. [Weishampel and Heinrich, 1992, (22); Sereno 1999, 4(69); Liu, 2004, (156)]

191 Maxillary crowns, anteroposterior width: broader (0), approximately equal (1), or narrower (2) than dentary crowns. [Sereno, 1986; 1999, 3(25); Norman, 2002, (34); Liu, 2004, (157)]additive characters

192 Maxillary/dentary teeth, marginal ornamentation: serrations (0); denticles (1). [Sereno, 1999, 1(21); Liu, 2004, (165)]

193 Maxillary/dentary teeth, marginal ornamentations: fine serrations set at right angles to the margin of the tooth (0); coarse denticles angle upwards at 45◦ from the margin of the tooth (1). [Butler et al., 2008, (116)]

194 Marginal denticles. simple, tongue-shaped (0); curved, mammillated ledge (1), absent or reduced to small papillae (2). [Sereno, 1999, 3(18); Norman, 2002, (31); Liu, 2004, (166)]

195 Enamel on crowns of maxillary and dentary teeth, distribution: symmetrical (0); asymmetrical, thin veneer labially, thick lingually (1); restricted to lateral/medial sides in maxillary/dentary teeth (2). [Gauthier, 1986; Sereno, 1986; 1999, 1(56), 3(35); 4(87); Liu, 2004, (174)] additive character

196 Cervical number: seven/eight or less (0); nine (1); ten or more (2). [Butler et al., 2008, (135) ]additive character

197 Fusion between the intercentrum of the atlas and the neural arches: absent (0); present (1). [Butler et al., 2008, (132)]

198 Cervicals 1-3, vertebral articulations: free (0); fused (1). [Sereno, 2000: 42]

199 Atlatal neural arches, median contact above neural canal: absent (0); present (1). [Sereno, 1986; 1999, 2(68); Liu, 2004, (177)]

200 Epipophyses on anterior (postaxial) cervicals: present (0); absent (1). [Butler et al., 2008, (133)]

201 Axial neural spine, anterior extension of anterior process: anterior tip extending as far as (0), or considerably beyond (1), the prezygapophyses. [Sereno, 1999, 3(51); Liu, 2004, (181)]

202 Cervicals 4–9, form of central surfaces: amphicoelous or amphiplatyan (0); at least slightly opisthocoelous (1). [Butler et al., 2008, (134) ]

203 Postaxial cervicals, form of postzygapophyses: weakly (0), or strongly (1), arched. [Sereno, 1986; 1999, 3(37); Liu, 2004, (182)]

204 Prolonged middle cervical centra (posterior centra reduced): absent (0); present (1). [Gauthier, 1986; Liu, 2004 (184)]

205 Postaxial cervicals, neural spine height (above zygapophyses): prominent (0); rudimentary (1). [Sereno, 1986; 1999, 3(36); Liu, 2004, (185)]

206 Mid-cervical rib form: long, greater (0); or short, less (1) than length of two centra; Liu, 2004, (188)]

207 Dorsal vertebrae, number: 12–13 (0); 15 (1); 16 or more (2). [Butler et al., 2008, (137)]additive character

208 Articulation between the zygapophyses of dorsal vertebrae: 0, flat; 1, tongue-and-groove. [Butler et al., 2008, (136)]

209 Dorsal neural arch pedicel, height: less (0), or 150% or more (1), than centrum height. [Sereno, 1986; 1999, 2(31); Liu, 2004, (191)]

210 #Mid-dorsal neural spines: short and rectangular, height and length very similar (0), height more than twice length (1), [Norman, 2002, (41); Liu, 2004, (192)]additive character

211 Mid-dorsal transverse processes, orientation: less (0), or more (1), than 40° above the horizontal. [Liu, 2004 (193)]

212 Anterior dorsal neural canal, size: less (0), or equal or more (1), than 50% of the dorsoventral diameter of the centrum. [Sereno, 1986; 1999, 2(33); Liu, 2004, (194)]

213 Sacrals, number: two (0); three (1); four/five (2); six or more (3). [Butler et al., 2008, (138)]additive character

214 Posterior sacral ribs are considerably longer than anterior sacral ribs: absent (0); present (1). [Butler et al., 2008, (140)]

215 Three sacrodorsals with long ribs attaching to the ventral aspect of the iliac preacetabular process: absent (0); present (1). [Sereno, 1986; 1999, 2(69); Liu, 2004, (196)]

216 Sacral rib shape: subrectangular (0); strap-shaped (1). [Sereno, 1999, 4(4); Liu, 2004, (199)]

217 #Anterior caudal vertebrae, length of transverse processes relative to neural spine height: subequal (0); longer than neural spine (1); shorter than neural spine (2). [Butler et al., 2008, (141)]

218 Proximal caudal neural spines: height the same or less than 150% of the centrum (0); more than 150% of the centrum (1). [Butler et al., 2008, (142)]

219 Elongate tail (58 or more caudals): absent (0); present (1). [Butler et al., 2008, (143)]

220 Caudal neural spines, height: shorter (0), or longer (1), than respective chevrons. [Sereno, 1986; 1999, 3(91); Liu, 2004, (200)]

221 Anterior caudal neural spine end, shape: transversely (0), or anteroposteriorly (1), flattened. [Sereno, 1999, 2(34); Liu, 2004, (202)]

222 Distal caudal centra, proportions: length greater than (0), or subequal to (1), height. [Sereno, 1999, 2(35); Liu, 2004, (203)]

223 Distal chevron shape, mutual contact: tongue-shaped, isolated (0); inverted T-shape, ends in contact (1).[Sereno, 1986; 1999, 2(71); Xu et al., 2002, (84); Liu, 2004, (204)]

224 Tail club composed of 2 pairs of large ossifications (anterior pair largest): absent (0); present (1). [Sereno, 1986; 1999, 2(98); Liu, 2004, (205)]

225 Gastralia: present (0); absent (1). [Gauthier, 1986; Sereno, 1999, 1(51); Liu, 2004, (206)]

226 Epaxial ossified tendons present along vertebral column: absent (0); present (1). [Butler et al., 2008, (216)]

227 Ossified hypaxial tendons, present on caudal vertebrae: 0, absent; 1, present. [Butler et al., 2008, (217)]

228 Ossified tendons, arrangement: longitudinally arranged (0); basket-like arrangement of fusiform tendons in caudal region (1); double-layered lattice (2). [Butler et al., 2008, (218)]

229 Parasagittal row of dermal osteoderms on the dorsum of the body: absent (0); present (1). [Butler et al., 2008, (219)]

230 Lateral row of keeled dermal osteoderms on the dorsum of the body: absent (0); present (1). [Butler et al., 2008, (220)]

231 Mosaic of small osteoderms between larger osteoderms and on the ventral surfces of the neck, trunk, and proximal portions of the limbs: absent (0); present (1). [Sereno, 1986; 1999, 2(78); Liu, 2004, (212)]

232 U-shaped cervical/pectoral collars composed of contiguous keeled osteoderms: 0, absent; 1, present. [Butler et al., 2008, (221)]

233 Parascapular spine: absent (0); present (1). [Sereno, 1986; 1999, 2(47); Liu, 2004, (214)]

234 Sternal segments of the anterior dorsal ribs: unossified (0); ossified (1). [Butler et al., 2008, (145)]

235 Sternal plate, shape: kidney-shaped (0); shafted or hatchet-shaped (rod-like posterolateral process, expanded anterior end) (1). [Butler et al., 2008, (148) ]

236 Ossified clavicles: absent (0); present (1). [Butler et al., 2008, (147)]

237 Proportions of humerus and scapula: scapula longer or subequal to the humerus (0); humerus substantially longer than the scapula (1). [Butler et al., 2008, (149)]

238 Scapula blade, length relative to minimum width: elongate and strap-like, length is at least 9 times the minimum width (0); relatively short and broad, length is 5-8 times minimum width(1); quite wide length is less than 5 times minimum width (2). [Butler et al., 2008, (150)]additive character

239 Scapula acromion shape: weakly developed or absent (0); well-developed spine-like (1). [Butler et al., 2008, (151) ]

240 Scapula, blade-shape: strongly expanded distally (0); weakly expanded, near parallel-sided (1). [Butler et al., 2008, (152) ]

241 Fusion of scapula and coracoid: absent (0); present (1). [Liu, 2004, (218)]

242 Scapula in sagittal view: distinctly curved (0); relatively flat (1). [Xu et al., 2002, (86); Liu, 2004, (220)]

243 Scapular acromion, orientation: coplanar with blade (0); everted (1). [Sereno, 1999, 2(73); Liu, 2004, (221)]

244 Scapular blade, form of dorsal margin: relatively straight (0); strongly convex (1). [Sereno, 1986; 1999, 3(92); Liu, 2004, (223)]

245 #Coracoid shape: subrectangular (0); subquadrate (1); subcircular (2). [Sereno, 1986; 1999, 2(88); Liu, 2004, (224)]

246 Limb proportions, relative length of forelimb and hindlimb: forelimb about 50% or less (0) or 65% or more (1) than that of hindlimb. [Xu et al., 2006, (70)]

247 Humeral-femoral length ratio: more than 0.6 (0); less than 0.6 (1). [Butler et al., 2008: (153);]

248 #Deltopectoral crest development: well developed, projects anteriorly as a distinct flange (0); rudimentary, is at most a thickening on the anterolateral margin of the humerus (1); round, project medially as a distinct flange (2). [Butler et al., 2008, (154)]

249 Manual length (measured along digit 2 or 3, whichever is longest) as a percentage of the combined length of the humerus and radius: less than 40% (0); more than 40% (1). [Butler et al., 2008, (156)]

250 Humeral shaft form, in anterior or posterior view: relatively straight (0); strongly bowed laterally along length (1). [Butler et al., 2008, (155)]

251 Triceps tubercle on humerus: subtle or absent (0); prominent with descending ridge (1). [Sereno, 1999, 2(38); Liu, 2004, (228)]

252 Deltopectoral crest, length: 30% or less (0), or 35 percent or more (1), of humeral length. [Sereno, 1999, 1(8); Liu, 2004, (229)]

253 Proximal carpals, form: ovoid (0); block-shaped (1). [Sereno, 1999, 2(39); Liu, 2004, (233)]

254 Intermedium-ulnare articulation: free (0); fused (1). [Sereno, 1999, 2(40); Liu, 2004, (234)]

255 Carpals and metacarpal I, articulation: free (0); coossified as two blocks (1). [Sereno, 1986; 1999, 3(39); Norman, 1990; Liu, 2004, (235)]

256 Metacarpals with block-like proximal ends: absent (0); present (1). [Butler et al., 2008, (157)]

257 Metacarpal I length: more (0), or less (1), than 50% of metacarpal II length. [Sereno, 1986; 1999, 3(42); Liu, 2004, (238)]

258 Metacarpal II length: subequal to (0), or 70-80% of (1), metacarpal III length. [Sereno, 1999, 3(73); Liu, 2004, (241)]

259 Metacarpals V length: substantially shorter than (0), or subequal to (1), metacarpal III. [Sereno, 1999, 2(6); Liu, 2004, (240)]

260 Metacarpal I shape: normal elongate bone (0), widen or shorten, block-like (1), absent (2). [Norman, 2002, (49); Liu, 2004, (243)]additive character

261 Extensor pits on the dorsal surface of the distal end of metacarpals and manual phalanges: 0, absent or poorly developed; 1, deep, well-developed. [Butler et al., 2008, (162)]

262 Axis of manual digit I, orientation from the axis of digit III: less than 25° (0), 45° (1), or 60° or more (2). [Sereno, 1986; 1999, 3(41); Liu, 2004, (244)]additive characters

263 Manual digit I, phalanx 1 length: shorter (0), or longer (1), than metacarpal I. [Sereno, 1999, 1(70); Liu, 2004, (245)]

264 Manual digit I-phalanx 1, shape: longer than broad (0); broader than long (1).[Liu, 2004, (246)]

265 Manual digit I ungual, shape: claw-shaped (0); subconical (1); absent (2). [Sereno, 1986; 1999, 3(44); Norman, 2002, (52); Liu, 2004, (252)]

266 Manual digit III, phalangeal number: 4 (0); 3 (1); 2 (2). [Sereno, 1986; 1999, 3(20); Liu, 2004, (247)] additive character

267 Manual digits II-IV: first phalanx relatively short compared to second phalanx(0); first phalanx more than twice the length of the second phalanx (1). [Butler et al., 2008, (161)]

268 Penultimate phalanx of the second and third fingers: shorter than first phalanx (0); longer than the first phalanx (1). [Butler et al., 2008, (159)]

269 Manual digits II and III, ungual shape: claw-shaped (0); hoof-shaped. [Sereno, 1986; 1999, 2(7), 3(78); Norman, 2002, (53); Liu, 2004, (253)]

270 Manual digit II-ungual, shape: broader (0), or narrower (1), than manual digit III-ungual. [Sereno, 1986; 1999, 3(75); Liu, 2004, (254)]

271 Manual digit IV-ungual: present (0); absent (0). [ Liu, 2004, (255)]

272 Manual digit V, phalangeal number: 0 (0); 1 (1); 2 (2); 3 or more (3).[ Liu, 2004, (249)]additive character

273 Manual digit V-phalanx 1, length: less than (0), or subequal to (1), half the length of metacarpal V. [Sereno, 1999, 3(77); Liu, 2004, (250)]

274 Manual unguals strongly recurved with prominent flexor tubercle: absent (0); present.(1). [Butler et al., 2008, (163)]

275 Acetabulum: at least a small perforation (0); completely closed (1). [Butler et al., 2008, (164)]

276 #Iliac blade, dorsal margin: concave (0); horizontal (1); convex (2), convex but concave in posterior part (3). [Liu, 2004, (266)]

277 Preacetabular process, shape/length: short, tab shaped, distal end is posterior to pubic peduncle (0); elongate, strap-shaped, distal end is anterior to pubic peduncle (1). [Butler et al., 2008, (165)]

278 Preacetabular process, length: equal or less than 50% of the length of the ilium (0); more than 50% of the length of the ilium (1). [Butler et al., 2008, (166)]

279 Preacetabular process, lateral deflection: 0, 10–20° from midline; 1, more than 30°. [Butler et al., 2008, (167)]

280 Iliac preacetabular process, length: shorter than (0); subequal to (1), or longer than (2), the postacetabular process. [Liu, 2004, (264)]

281 Preacetabular process, distal end expanded: absent (0); present (1). [Sereno, 1999, 4(6); Liu, 2004, (265)]

282 Dorsal margin of preacetabular process and dorsal margin of ilium above acetabulum: narrow, not transversely expanded (0); dorsal margin is transversely expanded to form a narrow shelf (1). [Butler et al., 2008, (168) ]

283 In dorsal view, the preacetabular process of the ilium expands mediolaterally towards its distal end: absent (0); present (1). [Butler et al., 2008, (169) ]

284 Subtriangular process extending medially from the dorsal margin of the iliac blade: absent (0); present (1). [Butler et al., 2008, (171) ]

285 Subtriangular process, form and position: short and tab-like, above acetabulum (0); elongate and flange-like, on postacetabular process (0). [Butler et al., 2008, (172) ]

286 Brevis shelf and fossa: fossa faces ventrolaterally and shelf is near vertical and visible in lateral view along entire length (0), creating a deep postacetabular portion; fossa faces ventrally and posterior of shelf portion cannot be seen in lateral view (1). [Butler et al., 2008, (173) ]

287 Ilium, eversion of dorsal margin of postacetabular process: absent or weak (0) or prominent (1). [Xu et al., 2006,(92)]

288 Length of the postacetabular process as a percentage of the total length of the ilium: 20% or less (0); 25–35% (1); more than 35% (2). [Butler et al., 2008, (174) ] additive character

289 Medioventral acetabular flange of ilium, partially closes the acetabulum: present (0); absent (1). [Butler et al., 2008, (175) ]

290 Supra-acetabular ‘crest’ or ‘flange’: present (0); absent (1). [Butler et al., 2008, (176) ]

291 Ischial peduncle of the ilium: projects ventrally (0); broadly swollen, projects ventrolaterally (1). [Butler et al., 2008, (177) ]

292 Iliac pubic peduncle, shape (lateral view): tapers to stout, subrectangular process (0); subtriangular (1). [Sereno, 1999, 3(93); Liu, 2004, (269)]

293 #Pubic process of ischium, shape: mediolaterally compressed (0); rostrocaudally compressed (1). [Butler et al., 2008, (179) ]

294 Pubis peduncle of ischium: short (0); long, contacting pubis peduncle of ilium (1). [Maryańska and Osmólska, 1985, (30); Liu, 2004, (290)]

295 Ischium, shape of shaft: relatively straight (0); gently curved along length (1) Distinct bend at midlength (2). [Butler et al., 2008, (180)]

296 Ischial shaft, cross-section: compressed mediolaterally (0); subcircular and bar-like (1). [Butler et al., 2008, (181)]

297 Ischial shaft is broadest at mid length and tapers proximally and distally: absent (0); present (1)

298 Ischial shaft: expands weakly, or is parallel-sided, distally (0); distally expanded into a distinct ‘foot’ (1); tapers distally (2). [Butler et al., 2008, (182)]

299 Groove on the dorsal margin of the ischium: absent (0); present (1). [Butler et al., 2008, (183)]

300 Tab-shaped obturator process on ischium: absent (0); 1, present (1). [Butler et al., 2008, (184)]

301 Ischial obturator process: distal (0); or near pubic peduncle (1). [Maryańska and Osmólska, 1985, (18); Gauthier, 1986; Sereno, 1986; Norman, 1990; Weishampel and Heinrich, 1992, (35); Sereno, 1999, 1(94), 3(11), 3(29); Liu, 2004, (294)] in the middle of the shaft (2)

302 Ischial symphysis, length: ischium forms a median symphysis with the opposing blade along at least 50% of its length (0); ischial symphysis present distally only (1). [Butler et al., 2008, (185)]

303 Pubis, orientation: anteroventral (0); rotated posteroventrally to lie alongside the ischium (opisthopubic) (1). [Butler et al., 2008, (186)]

304 Shaft of pubis (postpubis), shape in cross-section: blade-shaped (0); 1, rod-shaped (1). [Butler et al., 2008, (187)]

305 Shaft of pubis (postpubis), length: approximately equal in length to the ischium (0); reduced, very short or absent (1). [Butler et al., 2008, 2011: (188)]

306 Body of pubis, size: relatively large, makes substantial contribution to the margin of the acetabulum (0); reduced in size, rudimentary, nearly excluded from the acetabulum (1). [Butler et al., 2008, (190)]

307 Body of the pubis, massive and dorsolaterally rotated so that obturator foramen is obscured in lateral view: absent (0); present (1). [Butler et al., 2008, (191)]

308 Pubic acetabular surface, orientation: dorsolateral (0); lateral (1). [Sereno, 1986; 1999, 2(42); Liu, 2004, (280)]

309 Prepubic process: absent (0); present (1). [Butler et al., 2008, (192)]

310 Anterior pubic blade. Blade-like, unexpanded distally (0), blade with constricted proximal portion followed by a distal expansion (1), short constriction and deeply expanded (2). [Norman, 2002, (58)]; Liu, 2004, (282)]

311 #Prepubic process: Compressed mediolaterally, dorsoventral height exceeds mediolateral width (0); Rod-like, mediolateral width exceeds dorsoventral height (1); Dorsoventrally compressed (2); twisted along length dorsoventrally compressed at its base and transversely compressed distally (3); rod-like, twisted along length-mediolaterally compressed at its base whereas the lateral surface faces dorsally at its cranial end (4). [Butler et al., 2008, (193); Han et al., 2012; Ruiz-Omeñaca et al., 2012]

312 Prepubic process, extends beyond distal end of preacetabular process of ilium: absent (0); 1, present (1). [Butler et al., 2008, (194)]

313 The angle between prepubic process and pubic shaft: greater (0), or less than 150° (1). [Weishampel and Heinrich, 1992, (32); Liu, 2004, (285)]

314 Obturator opening in pubis, form: foramen (0); notch (1). [Sereno, 1986; 1999, 1(40); Liu, 2004, (288)]

315 Femoral shape in medial/lateral view: 0, bowed anteriorly along length; 1, straight. [Butler et al., 2008, (197)]

316 Femur length: shorter (0); longer (1) than tibia length. [Weishampel et al., 2003]

317 Femoral greater trochanter: small (0); broader anteroposteriorly (the width greater than head) (1). [Maryańska and Osmólska, 1985, (17); Liu, 2004, (296)]

318 Femoral head: confluent with greater trochanter, fossa trochanteris is groove-like (0); fossa trochanteris is modified into distinct constriction separating head and greater trochanter (1). [Butler et al., 2008, (198) ]

319 ‘Anterior’ or ‘lesser’ trochanter, morphology: absent (0); trochanteric shelf ending in a small, pointed, spike (1); broadened, prominent, ‘wing’ or ‘blade’ shaped, subequal in anteroposterior width to greater trochanter (2); reduced anteroposterior width, closely appressed to the expanded greater trochanter (3). [Butler et al., 2008, (199)]

320 Level of most proximal point of anterior trochanter relative to level of proximal femoral head: anterior trochanter is positioned distally on the shaft, and separated from‘dorsolateral’ trochanter/greater trochanter by deep notch visible in medial or lateral view (0); anterior trochanter positioned proximally, approaches level of proximal surface of femoral head, closely appressed to ‘dorsolateral’/greater trochanter (no notch visible in medial view) (1). [Butler et al., 2008, (200)]

321 Fourth trochanter of femur, shape: low eminence, or absent (0); prominent ridge (1); pendent (2). [Butler et al., 2008, (201)]

322 Fourth trochanter, position: located entirely on proximal half of femur (0); positioned at midlength, or distal to midlength (1). [Butler et al., 2008, (202) ]

323 Anterior (extensor) intercondylar groove on distal end of femur: absent (0); present (1). [Butler et al., 2008, (203) ]

324 Posterior (flexor) intercondylar groove of the femur: fully open (0); medial condyle inflated laterally, partially covers opening of flexor groove (1). [Butler et al., 2008, (204)]

325 Tibial posteromedial flange, lateral extension: does not reach fibula (0); extends posterior to medial margin of fibula (1); extends posterior to entire distal end of fibula and calcaneum (2). [Sereno, 1999, 1(45); Liu, 2004, (305)]

326 Cnemial crest on tibia: absent (0); present (1). [Benton, 1999, (46); Sereno, 1999, 1(13); Liu, 2004, (306)]

327 Fibular facet on the lateral margin of the proximal surface of the astragalus: large (0); reduced to small articulation (1). [Butler et al., 2008, (207)]

328 Calcaneum, proximal surface: 0, facet for tibia absent; 1, well-developed facet for tibia present. [Butler et al., 2008, (208)]

329 Distal tarsals 3 and 4: present (0); absent (1). [Sereno, 1986; 1999, 3(96); Liu, 2004, (312)]

330 Medial distal tarsal: articulates distally with metatarsal III only (0); articulates distally with metatarsals II and III. [Butler et al., 2008, (209) ]

331 Metatarsal arrangement: compact, closely appressed to one another along 50–70% of their length, spread distally (0); contact each other only at proximal ends, spread strongly outwards distally (1). [Butler et al., 2008, (210)]

332 #Digit I: metatarsal I robust and well-developed, distal end of phalanx I - 1 projects beyond the distal end of metatarsal I (0); metatarsal I reduced and proximally splint-like, end of phalanx I - 1 does not extend beyond the end of metatarsal II (1); metatarsal 1 reduced to a vestigal splint or absent, does not bear digits (2). [Butler et al., 2008, (211)]

333 Metatarsal III length: more (0), or less (1), than 30% of femoral length. [Sereno, 1986; 1999, 2(28); Liu, 2004, (317)]

334 Metatarsal IV shaft axis, curvature (anterior view): straight (0); sigmoid (1). [Sereno, 1999, 1(18); Liu, 2004, (318)]

335 Metatarsal V, length: more than 50% of metatarsal 3 (0); less than 25% of metatarsal 3 (1). [Butler et al., 2008, (213) ]

336 Phalanges: elongate (0); short but uncompressed (1), compressed (2).[ Liu, 2004, (320)]

337 Pedal digits II-IV, ungual shape: claw-shaped (0); hoof-shaped (1). [Sereno, 1999, 2(7), 3(60); Norman, 2002, (67); Xu et al., 2002, (98); Liu, 2004 (326)]

338 Pedal digit I, phalanx: present (0); absent (1). [Sereno, 1986; 1999, 2(44), 3(97); Liu, 2004, (321)]

339 Pedal digit III phalangeal number: 4 (0); 3 or fewer (1). [Sereno, 1986; 1999, 2(45); Liu, 2004 (322)]

340 Pedal digit IV phalangeal number: five (0); 1, four or fewer (1). [Butler et al., 2008, (212)]

341 Pedal digit V phalanges: present (0); absent (1). [Gauthier 1986; Sereno, 1986; Benton, 1999, (71); Sereno, 1999, 1(47), 3(82); Liu, 2004 (324)]

342 Head of humerus is separated from prominent medial tubercle on proximal surface by a groove: absent (0); present (1). [Butler et al., 2011: 223]

343 Pendent fourth trochanter, rod-like with subparallel anterior and posterior surfaces: absent (0); present (1). [Butler et al., 2011: 224]

344 Fibula, distal end is strongly reduced and splint-like: absent (0); present (1). [Butler et al., 2011: 225]

345 Astragalus and calcaneum are indistinguishably fused to one another: absent (0); present (1). [Butler et al., 2011: 226]

346 Maximum expansion of distal tibia relative to proximal: Distal tibia is considerably less expanded than proximal (0); Maximum expansion of distal tibia is subequal to that of proximal tibia (1) [Butler et al., 2011: 227]

**Table A**. Codings for S1 phylogenetic analysis, in TNT format.

*Euparkeria* 0100--00000000000021000000000010000000----010000000000000?00000000000000000-0010000000010010000-0--000000000?0000??00000000?000000-0--------0-00000010-0--?0000000?000002000000--00--010000001000-0000011000001000000000000000?0000-1?0000-1011100000?00?000??0000?10?00??????00??120000-000--?2010-0-000000-00000000----001?00-00000000000000000000000000

*Herrerasaurus* 0100--00000000000121000000010000000000----0100000000100000000000000000??000-00101010000100?0000-0--000000001??000???10001?0?000000-0--------0-00000010-0--?0000000?000002-00000--00--010000001000-0?00010001001000000000000?0000000-0?0000???0110000200010011-010000101000010000-11100000000-001000000000000-00000000----001001010000100000001000000010000

*Abrictosaurus*

?100--?11?01??1?00???????010?01?000??10000?????????????????????1???000??????????????????????????0-???????????????????????????00000-11?001---0?000100?101000????00??010?030000100-0?--?001101111110?????1??001????????????????????10-00000?????1???????00?00100010000?010????????0?021002?000-1111100??????????????????????000?20200021????01011000001?01??

*Tianyulong*

???0--11?001011?00?00?00?110?0???000??????01???????????????????????????????????????????????????????????????????????????????????????11?000?--?0100100?10100001??0?1?0000040010100-1?--1010101101110??????????????00?????????????0?1100000?????????????0001001??0?00?010100001000?010???????????????????1000?0-?11???????????0??????????????01??100000???1??

*Heterodontosaurus* 0100--11110111110010010001101010010001000001000101001000010100010000001002010020101010011000000-0--0000000021000000010001000000000-111001---00100100110100001100000010003001010111100101111111111011?0?00000010000003000000000101100000000?0001000010000100100010000101000010002010210021000-101110000100000-11100001000000000212000211?000101100000111110

*Lesothosaurus* 0100--00100000110010000000100010000101000000000000001000001000000000000000010000101000001010000-0--0000000011000000010001000000000-1010000001000000000-1000010001000100000100100-00--00001111011100?0000000?1???????2???????00?0111000000???0110000??010000???00000000000?000??1000210020000-001000000000010-011000010000100002020002111?00101100?0??00001

*Stormbergia*

??????????????????????????????????????????????????????????????????????????????????????????????????????????????????????????????????????????????????????????????????????????????????????????????????????????0?1??0?00?????00??00?0?1??000??????11000010?????????????????????????????0110020000-00101000000001101110000100?10&1000020?0002111??0?01?000?????001

*Agilisaurus* 0100--0110000011000001000010000??1000100011-0000010020000000000000100000011001?0001000001010000-0--00?000001000010001000??0??00000-10?0?000?0000000001010001-0000000100011120100-00--10201111011100100?1?10011100000200000000010110000000???111000010012?001??????????????????????0210020000-00100000010001100110000101100000?20200021111?0101100000100001

*Yandusaurus*

?????????????????????????01000?0010??????????0000000?0?00????????????0?0??????????????0??????????????????????????????????????00000-?????????????????0???????????????????????0101100-?10??11110?11?1??????00011?00100????????0??01?????0?0???011000010??2?101???0????????????0????0??????????????????????????????????????????1130??0021??????0??00????0????

*Haya* 0100--001?10001?0010010?0110000??1000101001-00000000100000110000101000000?1000?0011000001010000-0--00000000100??00001000????000000-11100010011000000?1010?01-00000?00000101001011000-1000111101110?10001?000110000003???00?00?1011010000000?0110000100000001000000?0????0?????????0210020000-1011110000000?100110000100111001131200021?1110001?00000100001

*Hexinlusaurus* 0?00--0?????0????????10???10001??10?0100001-0000000020000011000000?000000??0???0?0100000101?000-0--000000001?????????????????00000-?????????1??0000001010??????????0000?????0101000-0100011110?1100100010000111000002000000000101100000000?0111000010000000100000000000000000??2000110?20000-1010100000000010111000010110000102020002111110101100000100001

*Changchunsaurus*

?1?0--001?10?011001001000110021??100010??01-00011000100000011?00001000000?1-?0?0???0??00????????0--?0000????00?0000010?0??0?000000-1110001001100010001010001-1000000100010100101100--1000111101110??00?1?000111???003??????????011??0000000??1100?010??0?001??????????????????????0???????????????1????????????????????????01131?????1????00?1?0000?10??0?

*Hypsilophodon* 0100--0010100011000000000110101001000101001-000000002000000100001010000002100000011000001010000-0--000000001100010001000100?000000-11100010?1000000001010001-10000001000101001010010-10001111011101100010000111000003000000000101110000001000110000100000001000000000000000000?2-00210020000-10111100000000101110000101110&100113120002111110001100000100001

*Jeholosaurus* 0100--1010100011001001000110001??1000100001-000010001000000&1100001010000000100000011000001010000-0--00000000100?000001000?10?000000-11100000010000100?1010001-00000?0000000100101100--?000111101110?10001?0001??00000300000?00010110000000??0?1??000??000?001??????????????????????0210020000-101111000000011001100001???1100113120002111110101100000100001

*Gongbusaurus* 0100--0010?0001100?001000?10001??1010100001-00000000100000?11000001000000?10000?011000001010000-0--?00000001000000?01?00100?000000-10100010010000100?0010001-00000?0000010110100100--?00011110?110??????0000???00000300?00?00?1011?000000??00110000100000001??????????????????????02100?0000-1011110000000?100110000100111001?3120002111?1010110000010000?

*Gideonmantellia*

???????????????????????????????????????????????????????????????????????????????????????????????????????????????????????????????????????????????????????????????????????????????????????????????????????????????00?00????00?00010????000???????????????????????????????????????????011?????00-00?1110???????????1????114???001130?000?111??0?01?00000????01

*Parksosaurus* 010???0????00????????0?????1001??100010??01-0?00000??0?0000?0000?0?0?0?0???-??00?1100000???0000-0--???00?0?????0?????????????00000-??????????100000001010??1??00?0?0100?????02??100--?000111101110????????????20????30??0?0???1?111000?0?10?02?0??????02??????????????????????????0?100??000-????1??0?000001?111?00?1?41??00??312100??????00??1????0??0001

*Thescelosaurus*

?10???????10????0??????????1???001???10101??0?000?00?0?00?1?000?????0?0????????-0110000?1????00-0--???000?1??0001????????????00000-1????????11000000010100?1-1?020?0?000??1??1??101001000111???1101??????0000110000?300001?000101110000001?00210000?0?0200010000000000000000??12000110020000-1?11110000000010111000010411101113121002111?10001100000100001

*Gasparinisaura* 0??????????00???????????0111000??1000100001--0000?00-000000?0?00000000001?1-00?0??100000?0?00?0-0--?00000?0?????????????????000000-?????????10000000?1?10001-?0000?0100?????010111?0-10001111011101?0????00?1???0000200?0?????1011100000???0?1000?0?0??0?00???????????????????????0310020000-1001?10000000010111000?1011100?11312000?11111010110010010000?

*Dryosaurus* 0000--11?100111110000?0?0111001011010100010000000000?100000100000?100000??000000001000001?10000-0--000000011?0001??01000100?000000-110100100110000000101?0?1-00000?01001----021101101110011111111011?00101001110000030000100001011000000000001000?01??02?001000??00???0?????????0?0110020000-1001110000101010111000011011001113020112111110101110100100001

*Zalmoxes*

?1?0--010?00011?00001000?100000001001?????1?000001003110010?000?0??00000121-00210010000?1000000-0--000000?0?00?01000???????????????1?00001000000000011111?01-10001001001----021110110100011110111011?00111101?2001003?00????0????1???????????1010?000??00?01??????????????????????0210020110--011111001000?0?1????????????111130211?2111??????????????????

*Tenontosaurus* 0100--01?0000111100010000101000011000101001-000000002010000100001010000?120000000010000010?0000-0--000000011?0001???100???0??00000-110100001110001000111?0?1?10020?01001----02110010011001111?111012000001100120000?20000110001011100000000001000?01??0000010000000000000110001?000310020000-1?11110000001010111000011011011113021112111110001110000100001

*Iguanodon* 0100--110000111110001000020---0011001100001-010001003100000100000110000010000000001000001010000-0--0000001?2100020001000200?000000-1101001011201010001111101-00000001001----1211011111100111112111120001111011200100300021000010110100000010010100000100010110111101021011101113100110020000-1101110001101011-11100012011111113021112111000101-20100100001

*Probactrosaurus* 01?0--?1000011111000100002??---??10?1?????--??00010?3?0000?10?000???0?001??0???00?10?001?010000-0-?0000????????????????0??????0000-11010010?12000100011111?1-?00???01001----1211011011100111?121112??0?????0????010?3?0021??00?01??100000010?10?0?0?0100??01????11?1?2?11110111??00110?20000-1101111000101011-1110?012011?111130211121????0101-2010010000?

*Ouranosaurus* 01?0--11000001111000100002000000110011000000?100010031000001000001??000010000??01?1000001010000-0--0000001?0??????????????????0000-110???101?2010100?11111?1-10000001001----1?11????11???????121?112?00?1?10112001003?0021?10?101??100000????10?0000010?00011?1?1101??111???1???1?0110?20000-1101111000101011-1110?0120111111?30211121??1?0101-2010010000?

*Camptosaurus* 0100--111??001111?0??000010---0?110??10000000000000031000011000000?000001?1-0??0001000011010000-0--00000001100002?101????????00000-??????????1000100?111100?-10000001001----02110110111001111121101100?1?11011?001002?0021?00010110100000???011000010102000110111101010111100012100110020000-1001110001101011?110000110111111?30211?21??100101-10000100001

*Stenopelix*

????????????????????????????????????????????????????????????????????????????????????????????????????????????????????????????????????????????????????????????????????????????????????????????????????????????????????31000???0?1?????00000????0?1???0??????????????????????????????0210021110--011100??2010?0-?1?????100???001?31??????????000??00000?????1

*Yinlong* 20111100100000100010010000110010010001011001200010000&1010000100100010000&1100010020101001011000000-10101001101100?0211010011000000000-10100011010?00000?101000011010110&1000031120100-00--1000111101110?10001?000111001003?000000001011000000????00?0000000000001000000?0000000000?????02100111100102110000001000-?1?1?0010100?001130200021?1??0001-000001?0001

*Chaoyangsaurus* 20?11??0??00??1001?1?????0??????011??????????00010???0100????????0?0001?00????2???????0?????????????????????????????1001?????00000-10000011010?0000011010001-?010111000040100100-000-10001111011100??001?0001???????????????????1?????0???????1?0?0????0??01??????????????????????????????????????????????????????????????????????????????????????????????

*Xuanhuaceratops*

??????????????10????????????????????????????????1?0???100?0?????0010001?00????2???????????????????????????????????????????????????????????????02??????????????0??111???05???01?0-0000?0??1?1?001100?????????????????3????????????????????????00?000??????1??????????????????????????????????????????1?????????????????????0?1131?0????????????????????????

*Liaoceratops* 21?11100?0001010001001000110011??10001011001000001112001000010100111001000010020101011011101000-1112110010110000110110011100000000-11101011010?000000101100??0100110000030120111111101000111100110??????????????????????????????1???0000??????????????????????????????????????????????????????????????????????????????????????????????????????????????????

*Yamaceratops* 2??111???????????????????11?011001000?????0100100101000110001010011110110???00?010101101?10??00-1???110?101?1??01?0110011?0?000000-1?100????11020011?1011011-?0011000000??1??1111111010001111001102????????????????????????1????????0000??????????????????????????????????????????021??????0--021110????????????????????????????????????????????0?????????

*Archaeoceratops*

211111101000?010001000000210011??100010110012000111?0010?00?10100111101002?00020101011011?????0-???21?0010?11?00????1001??0?000000-111010110100000000101100?-?1011010000301201111111010001111111101????1?00???010000310?????????????0000??????????????????????????????????????????0210010010-1021110??0000????11100010200???1131???????11?00?1-000001???0?

*Auroraceratops* 2111111010000010001000000111011??100010110012010111100111000101001?1101100010020101011011101100-1112110010111??0???1100?110?000000-111000110100001000101110011101101000030120111111101000111101110?211?1000011110100310021010110110000000001010000000000000100000000000000000?11000210010110-1021110?0110000-111100010200000113121002111??00?1?10000100001

*Helioceratops*

?????????????????????????????????1?????????????????????????????????????????????????????????????????????????????????????????????????1????????1100010001112?1?????????????????01111111010001111011101???????????????????????????????????????????????????????????????????????????????????????????????????????????????????????????????????????????????????????

*Koreaceratops*

?????????????????????????????????????????????????????????????????????????????????????????????????????????????????????????????????????????????????????????????????????????????????????????????????????????????????????????1010110??????????????????????????????????????????????????????????????????????0100???1??????????????????????2?111100??1100?????00?

*Protoceratops* 21111100100000100011011002110110?100?1011001201012012001100010100111101100000020101011011101100-1112100010?110?01??11001??0?000000-1110101101100011101011111-?00010000004011011111110100011111111022?1??10001?000100310021010100110000000??10100010001?0000110000000000000000012100210010000-102111000100000-111100010?00100113120002111110001111000100001

*Bagaceratops* 2111?100?0000010001100100211011??100?1011001201001010001100010100111101102000020101011011100100-11111000101110?01?011?01010?000000-111010?1011000100?1011101-??001000001----01111111010001111111102?????????????????3???????????????????0???????????????????????????????????????????????????????????0???????????????????????1?31??????????????????????????

*Leptoceratops* 21111100?0000010001000000111011??100?10??001201012010001100010100111101102??0020101011011100000-1112110010111??01??111011?0?000000-11101011011020101?1011111-?1001000001----011111110100011111111012?1??1000110001003???21?1010011000000000100??01001100000100000000010100000011000210010000-1021110001000?0-?11100010?001101131101021??1?0001110000100001

*Psittacosaurus* 2001001010001010012100010?0----00110010110--201000001001000100000011000000100020001001011100000-1010000010?0100000001101100?000000-10000001011000011?10110101?0100000001----01011110010001111011101?000100001100000030000?00-0101100100000010110010010000001000000-0000000000010-00210010000-?02111000000000-11110001020010011312010211111000110000010??01

*Hongshanosaurus* 21?100101000101001210001000----??110?10110--201000001001000100000011000000100020001000011100000-10100000101010?000001101100?000000-11000001011000011010110101?0100000001----01111?1001000111101110???0????????????????????????????????????????????????????????????????????????????????????????????????????????????????????????????????????????????????????

*Scelidosaurus* 0100--0??0?0001??????00?0?1100100100011--00?10000110200000001000000000100??-001110100?00?01?000-0--00?000?01?1000????00???1??10000-?????????1???000000-1?0?1-00010?001001?10?100-00--0000111101110000000?00?0?2000002000000000101100110100?001100000??00?001??????????????????????0110020100-000000000000000-1110010100001000020210021111?0001111000100001

*Scutellosaurus*

??00--??1????0?100???????????????00?????????10000?00?0000??????????0?0????????1????00????????????-???????????????????????????1???????????????0?0000000?????????????0?0?00??0?100-00--00?01111011100100???000??100?0?2???0?1?00?0?1??11000?????1?000?2?00?001???0????0?????0?0????00110020000--01?00?00?000????110000100?010000202100?1????0????000???00001

*Emausaurus* 01?0--00?00000?100200?000?11001001000100000010000100100000001000?00000??0??-???010100000???0?00-0--00?00?????0?00????????????10000-?????????1001000000-100?0000010?0000010100100-00--00001111011100??????????1????????????????10????110?0??????????????????1???0?????????????????????????????????????????????????????????1??????????????????????????1?????

*Minmi* 1???--????????????-???0???0-?--????????-???-????????????0?????-??-??0???0??-??1?-?1??-1-10?0????0-???????0?0?????????????????100210?????????????1?????-?00????????????0????????0????-????????0?1?00?????????????0010??10???00?101???11110?????????1??1????01??????????????????????11?1?201???0??00???0??????-????110?0??011?0?????????????11?????????0???1

*Gargoyleosaurus* 1??0--?0??????????-00?000?0-?--????????-???-????????????00?-??-??-??01-001?-??1?-????-00?010????0-?????????0???????????????00111221??????????0??1?????-?00??-?0??????10??0??????????-????????011?00?????????????????????????????????11?1?????????????1????????????????????????????1??1???1???0??00???????????????11???????1???????????????11?????????0???1

*Pinacosaurus* 11?0--011000001100-000?0110----??100011--2-?-00001-0-0?0000-00-00-?101--01101011-0110-1-101?010-0--?0?000010?1000??01000201111112111?00010??11001000?0-?00?1-00000?0010??-??0??0????-????????011?00??01?0?0011??001???10???000111???11110????2??111?1100?001??0?0011?001?1??1?????12?1?201???0??00???0??????-????11???????1?0???0???21????1111???0??10???1

*Euoplocephalus* 1-??--?11000?01100-000001?0----??10??11--???-00001-?-??0000-???00?0101--011010?1???10-1-??????0-0--00?00001001?0000010?0?0111111221?????1????1001?????-????1?????????1??????1???????????????????????101???????????????1????????1?????????????2?1?1?????0????????????????????????????????????????????????????????????????????????0??????????????21?????????

*Huayanggosaurus* 0100--0010100011001101000110001?0100011--2001000010020?000001000001001--01010111101000001010000-0--00?000001??000???1000?00?000000-1000000?010010000?10100?0100000?0000000101100-00--0000111101110?11??1000011??10112?00?1?101?011??110??????1?11001??0??01101???????????1????????0211120100-0000000?0????????11?0011000111100??0-0021??1?1111-21?1110??01

*Stegosaurus* 0100--00?000001110101000021000-00100011--20010000100200000000000001001-0011-0111001000011010000-0--00?00000111000??010000?10000000-1000000?012010000?0-10000000000?01001---?11101100000??1?1?0111002100100000120101120002101111011001100100?011101002100??1111000011000102?-1?11000211120100-00000000000020--11100111100111100300-0?21??111210-2111110??01

*Wannanosaurus*

??0?????????????????????????????????????-???00001?00???000001?2????????????????????00?0?111?0010100110010????????????????????00000-1????????00?00000?10000?1-00000?0000?????0110-00-?00??11110?110???????0??????????????????????????0000??????????????11?101?????????????????????????????1????????????????????????????????011?3111??2????????????????001??

*Homalocephale* 0?00-?????????????????0??????????10??11--1--000011102000000011200110001000100001001000001110011010011011000110110??11010??0?000000-??????????????????????????????????????????1?010?0??0??11110?1101????????????10000310110??00?011120000001???????????????????????????????????????02100111111-12111011100000-11111001131120??1??10?02?????0????0??????0101

*Goyocephale* 0??0--???0?1?0???????00????----??????11--1--???????????000?-?12????????????????10010000?111000101001??01???????10??1??????????0000-?????????00?00000?10?001?-?0000?10010301?01001000-002?1111011101?????????????????1??1????00?01?12??0?0?????????????11?101??????????????????????02100111110-1211??????????????????????????????????????110????00?????????

*Stegoceras* 01?0--00?000001100000000001----00100011--1--0000111020000000112001100010000100-10-11000011100111100110010001??110??11?10210?000000-?????????0??00000?10000?1-0010001001030100100100--002?1111011101????????????10000????10?000?0111100000????10?000?0?11?101??????????????????????021001?1111-121110111000?0-?????????????0?????100?2??????2???00??????1??

*Prenocephale*

00?0--001?0100110000000?011---000100011--11-0000111020000000112001100010001-0001--11--001110011110011011000100110?111010110?000000-????????????????????????????????????030?101?0??????02?11110?1101????????????1????31?11???????1?1???0?0??????????????????????????????????????????2100111111-12?110111000?0-?????????????0?1???10????????????????????????

*Ischiceratops*

????????????????????????????????????????????????????????????????????????????????????????????????????????????????????????????????????????????????????????????????????????????????????????????????????????????????????310021?10?0??100000???????????????????????????????????????????0210000000??021110??2011010?????????????101?3020??21????????????????00?1

*Zhuchengceratops*

??1????????????????????????????????????????????????????????????????????????????????????????????????????????????????1?1?????????????1??010????1020101?1011?????00?1??0?0?????01111111010001111111102????????????0?10???????????????????????????????????????????????????????????????????????????????????????????????????????????????????????????????????????

*Motanoceratops*

2111??10?0??0???00??001???11?????1???10??0????????0????1100????0011110110?????2?????????????????????11??1?1?1???2??????????????????1?1??????1??????1????1?????1??1???????????111111101000111111110?2?1??10001100010031002101010011??000????1?1??010?1?????????00000000000000001?000210010000?1021110001000?0??11100010200110113020102111??00011100001?0001

Ceratopsidae

211111000000011000110010010?0?1??100?101100?201012010001100010100111101100000020001010001010110-11121001101010?01??11101110?000000?1010101001100010001112101??1001000001????111110100100011111111020?1??100001000100310021?10100110100000001010100001100000100000000000000000012?00210010110?112111000110000?111100011200111113000102111111001121000100001

*Cerasinops*

2??1??????????????????????????????00????????20101?0?0001??0??0?001111011????0?201??0??0?1?0?000-??12??0?1?0?10?01???????????????????????????1?00010??1011?????1?11?????0?0???111???????0?????????????0?????????1010??????1?1?1001???00000????0??01???100?001??????????????????????0???????????0???????200000-?????????????1011302010211???01011100011000?1

*Udanoceratops*

???11100?00??01?0011??000?110?1??1????????002010?2??000?1????????1???01100????????????01???????????????????????????????????????????????????????2?1?1?????????????1?????????????????????????????????????????????0?????????1?1??????????????????????????????????????????????????????????????????????????????????????????????????????????????????????????????
